# Reconciling glacial Antarctic water stable isotopes with ice sheet topography and the isotopic paleothermometer

**DOI:** 10.1038/s41467-018-05430-y

**Published:** 2018-08-30

**Authors:** Martin Werner, Jean Jouzel, Valérie Masson-Delmotte, Gerrit Lohmann

**Affiliations:** 1 Alfred Wegener Institute, Helmholtz Centre for Polar and Marine Sciences, Bussestr. 24, 27570 Bremerhaven, Germany; 2 Laboratoire des Sciences du Climat et l’Environnement (LSCE), Institut Pierre Simon Laplace, CEA-CNRS-UVSQ, UMR 8212, Université Paris Saclay, 91191 Gif-sur-Yvette, France

## Abstract

Stable water isotope records from Antarctica are key for our understanding of Quaternary climate variations. However, the exact quantitative interpretation of these important climate proxy records in terms of surface temperature, ice sheet height and other climatic changes is still a matter of debate. Here we report results obtained with an atmospheric general circulation model equipped with water isotopes, run at a high-spatial horizontal resolution of one-by-one degree. Comparing different glacial maximum ice sheet reconstructions, a best model data match is achieved for the PMIP3 reconstruction. Reduced West Antarctic elevation changes between 400 and 800 m lead to further improved agreement with ice core data. Our modern and glacial climate simulations support the validity of the isotopic paleothermometer approach based on the use of present-day observations and reveal that a glacial ocean state as displayed in the GLAMAP reconstruction is suitable for capturing the observed glacial isotope changes in Antarctic ice cores.

## Abstract (web summary)

Despite their importance, the accuracy of the quantitative interpretation of Antarctic ice core stable water isotope records remains a matter of debate. Here, the authors use an isotope-enabled atmospheric general circulation model to test and validate the isotopic paleothermometer approach.

## Introduction

Antarctic ice cores provide a wealth of information on past atmospheric composition and climate^1–4^. For these polar ice cores, stable water isotopes (H_2_^18^O, HDO) belong to the key set of analysed climate proxy variables. Such stable water isotope records from the various Antarctic ice cores provide high-resolution information on climate variability at decadal to glacial–interglacial time scales^5,6^.

Since the 1960s, the relationships between Antarctic stable water isotopes and temperature have been explored using surface measurements^7^ as well as Rayleigh distillation models^8^ and atmospheric general circulation models equipped with stable water isotopes diagnostics^9–11^. The strong linear spatial relationship between annual mean surface air temperature and *δ*^18^O today^7,12^ with a slope of 0.8‰/°C between the *δ*^18^O and local temperatures is fully consistent with theoretical Rayleigh distillation^13^ and is well captured by atmospheric general circulation models equipped with stable water isotopes (isoGCM), despite model biases for temperature or precipitation in Antarctica^14,15^. This relationship has therefore been commonly applied to estimate past changes in temperature from Antarctic ice cores^2,5,16–18^.

However, the consistency between present-day observations, distillation theory and isoGCM simulations is no proof that past temperature changes should scale to past precipitation *δ*^18^O changes with the same quantitative relationship. Indeed, several processes may affect the temporal isotope–temperature relationship, such as changes in evaporation conditions, atmospheric transport pathways, changes in condensation vs. surface temperature associated with polar boundary layer processes, or changes in precipitation intermittency or seasonality. In Greenland, where alternative paleothermometry methods are available from ice cores, temporal slopes vary from 0.3 to 0.6‰/°C^19,20^, much lower than the local present-day spatial slope of about 0.7‰/°C^21^, an up to a factor 2 difference attributed to changes in precipitation seasonality as well as moisture sources and transport paths^20,22,23^.

In Antarctica, the importance of several processes that could distort the *δ*^18^O-temperature relationship has been explored using deuterium excess measurements^24–26^ as well as low-resolution isoGCMs using simulations performed for colder than present-day climate^14,27^, including water tagging^27,28^, or warmer than present day climate projections^29^. The conclusions from these studies were that under high CO_2_ and warmer than present conditions, there may be changes in temperature-precipitation covariance accounting for a smaller isotope–temperature slope and for varying isotope–temperature relationships for different ice core sites on the East Antarctic plateau^30^. Glacial isotope–temperature relationships appear rather homogeneous in the East Antarctic plateau and moisture-source effects had limited impacts on glacial–interglacial temperature reconstructions. However, these isoGCM studies were performed with low-resolution atmospheric models and were not based on the latest reconstructions of ice sheet, sea ice and ocean sea surface temperature changes. Processes associated with moisture transport may not be properly resolved using atmospheric models with a resolution of 200 × 200 km^15,31^. Moreover, recent ice core data from West Antarctica have expanded the documentation of glacial–interglacial changes in ice core *δ*^18^O and evidenced different amplitude and timing of changes in different areas^32^, challenging the homogeneity of isotopic changes initially reported in the East Antarctic plateau^33^, where regional amplitudes also vary through time^34^. These new findings motivate further investigations of glacial–interglacial changes in isotope–temperature relationships in Antarctica using isoGCMs with an increased spatial resolution.

For such isoGCM simulations of the climate of the last glacial maximum (LGM), major uncertainties in required boundary conditions are the prescribed sea surface temperature, sea ice extent and ice sheet topography, while orbital parameters and GHG are well known for the LGM period. Large uncertainties remain associated with the knowledge of glacial West Antarctic ice sheet topography, evidenced by the large spread of existing reconstructions^35–39^. Here, results of isoGCM simulations with prescribed different ice sheet reconstructions are compared with ice core data from West and East Antarctica. The deviation between simulated water stable isotopes and ice core records provides a constraint on the realism of LGM ice sheet reconstructions.

## Results

### Present-day climate

**Fig. 1:**
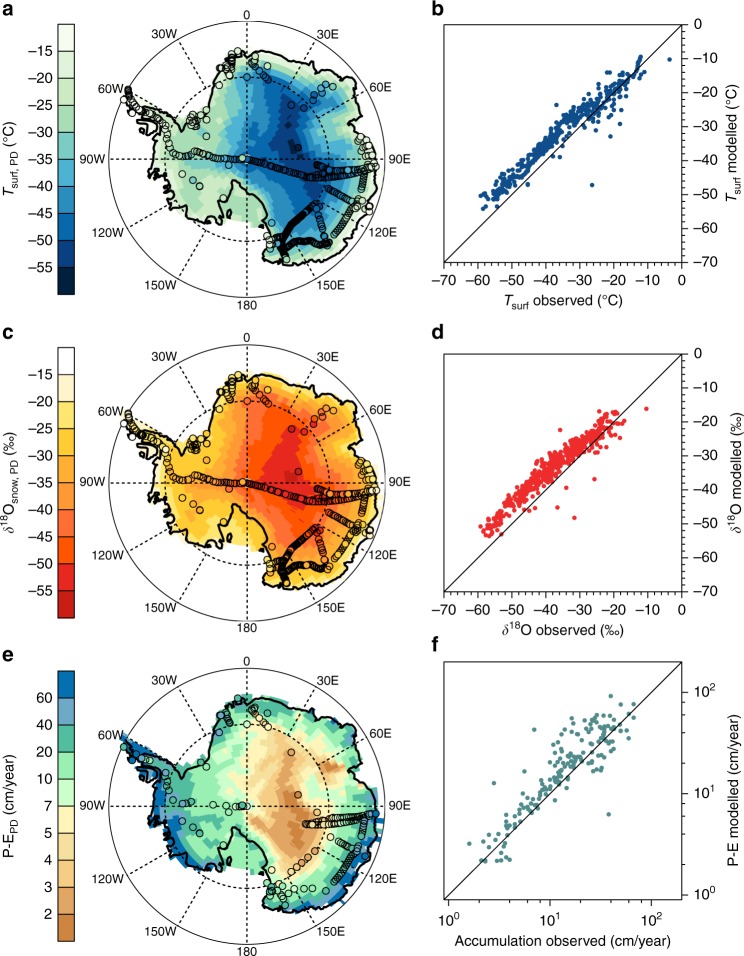
Comparison of present-day observational data and ECHAM5-wiso model results. **a** Map of present-day Antarctic surface temperatures as simulated by ECHAM5-wiso (background pattern) and observational data compiled by Masson-Delmotte et al.^12^ (filled circles). **b** Scatter plot of simulated Antarctic surface temperatures *T*_surf_ (*y*-axis) vs. observational data compiled by Masson-Delmotte et al.^12^ (*x*-axis). **c**, **d** Same as **a**, **b**, but for *δ*^18^O in surface snow. **e**, **f** Same as **a**, **b** but for accumulation rates. Please note the logarithmic axis scale for the accumulation scatter plot

As a reference simulation, the isoGCM ECHAM5-wiso has been run under present-day (PD) model boundary conditions with an observational-based distribution of ocean surface *δ*^18^O values (see Methods for details). This PD climate simulation shows a good agreement between observed and simulated Antarctic surface temperatures (*T*_surf_) with a correlation coefficient of 0.94 despite an overall warm bias, which grows from 0 to 5 °C in the temperature range of −20 to −35 °C (Fig. 1a, b). Such a warm bias over Antarctica is frequent in GCM simulations^40,41^. There is also a remarkable agreement between observed accumulation and simulated values, with a general wet model bias consistent with the warm bias (Fig. 1e, f). Simulated *δ*^18^O values in surface snow range between −18 and −55‰, and their distribution mimics that of the simulated temperature (Fig. 1c). Consistent with the model warm bias, *δ*^18^O simulated values are, on average, 5‰ less depleted that the measured isotope values, with modelled and measured *δ*^18^O values as strongly correlated as for temperature (*r* = 0.92; Fig. 1d). These comparisons illustrate that the ECHAM5-wiso model, despite its warm bias at very low temperatures, is able to capture the present-day Antarctic spatial isotope distribution in good agreement with available observations (see Supplementary Note 1 for details). This model skill for present-day is key to our confidence in the quality of the LGM simulations that we now examine.

### LGM climate

We have performed six different LGM simulations to evaluate the influence of different boundary conditions on the simulated temperature and isotope changes in Antarctica. The main focus has been put on recent glacial Antarctic ice sheet reconstructions. Furthermore, the influence of different prescribed sea surface temperatures as well as different patterns of the isotopic composition of the surface ocean has been investigated (see Methods).

In order to evaluate the quality of the different isotopic simulations, we have compiled *δ*^18^O data from the following 11 ice cores: Vostok, Dome F, Dome B, EDC, EDML, Taylor Dome, Talos Dome, Byrd, Siple Dome, Law Dome and WDC (Table 1 gives an overview about all ice core locations and isotope data). For this purpose, we expanded the comprehensive compilation recently provided by the WAIS Divide Project Members^32^ with data from Dome B^42^ and Taylor Dome^43^. We also investigate model results for sites where ice cores reaching back to the LGM have been recently obtained or will soon be drilled, but where isotopic data are not yet available (James Ross, Roosevelt Island, Berkner Island, Fletcher Promontory and Dome A). It is important to note that mean LGM values in Table 1 do not necessarily represent the most depleted isotope values at the end of the last glacial period. The onset of increase in stable water isotope levels at the end of the LGM occurred in West Antarctica at least 2000 years earlier than in East Antarctica^32^. However, as our idealised LGM simulation has been performed with boundary conditions of 21 ka B.P. (see Methods), we have chosen to use this identical time interval at the end of the last glacial from all isotope records as most appropriate for our model data comparison, using the synchronised Antarctic Ice Core Chronology 2012 (AICC2012^44^) for the Vostok, EDC, EDML and Talos Dome ice cores and the latest individual ice core chronologies for the other sites.

**Table 1:** Measured and modelled LGM-PD Δ*δ*^18^O changes for selected deep-drilling ice core sites

Site (sector)	Lat °S	Lon °E	Elevation (m a.s.l.)	Ice core Δ*δ*^18^O (‰)	Modelled Δ*δ*^18^O (‰)
EDML (1)	75.00	0.07	2892	−6.3	−6.2
Dome F (2)	77.32	39.70	3810	−4.9	−4.1
Dome B (3)	77.08	94.92	3650	−5.0	−3.6
Vostok (3)	78.47	106.87	3488	−4.8	−3.9
Law Dome (3)	66.73	112.83	1390	−5.5	−4.3
EDC (3)	75.10	123.35	3233	−5.6	−5.1
Taylor Dome (4)	77.80	158.72	2365	−4.0	−4.8
Talos (4)	72.82	159.18	2315	−5.5	−4.7
Siple Dome (5)	81.67	−148.82	621	−7.8	−8.4
Byrd (5)	80.02	−119.52	1530	−7.3	−9.0
WDC (5)	79.46	−112.14	1766	−7.3	−9.3
Dome A (2)	80.37	77.37	4093		−2.9
RICE (4)	79.36	−161.70	550		−2.8
Fletcher (6)	77.90	−82.61	873		−13.3
James Ross (6)	64.20	−57.69	1542		−7.2
Berkner (6)	79.55	−45.68	890		−15.2

The first four columns list the selected deep-drilling ice core sites and the sector they belong to, latitude, longitude and elevation. In the following two columns, the measured and modelled LGM-PD Δ*δ*^18^O for the different ice core sites are reported. Modelled values have been calculated for the PD and LGM reference simulations with prescribed PMIP3 ice sheet changes, and model results have been bilinearly interpolated from grid box values to exact latitude and longitude coordinates of each drilling location. No correction for glacial seawater change has been applied for ice core or modelled Δ*δ*^18^O values

Ice core data indicate non-uniform LGM *δ*^18^O changes over the Antarctic continent. The smallest change of −4‰ is found at Taylor Dome^43^. East Antarctic ice cores (Vostok, Dome F, Dome B, Law Dome, EDC and Talos) show LGM decreases of *δ*^18^O in the range of −4.8 to −5.6‰. A much stronger depletion in the range of −7.3 to −7.8‰ is reported for the West Antarctic sites of Byrd, Siple Dome and WDC. The LGM decrease at the EDML site (−6.3‰) is ranked in between the smaller East Antarctic and larger West Antarctic glacial changes. This change includes a correction for upstream effects at the EDML site, which is a source of uncertainty as it depends on glaciological modelling of past Antarctic ice sheet topography^25^.

**Fig. 2:**
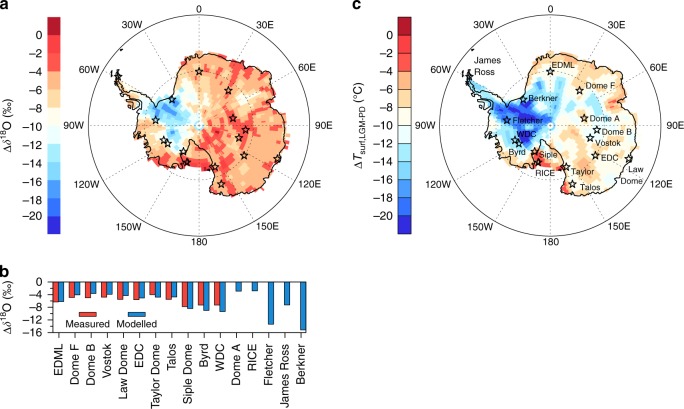
Simulated *δ*^18^O and temperature changes in Antarctica during the LGM. **a** Map of simulated LGM-PD change of *δ*^18^O in surface snow. Symbols mark the position of data from deep Antarctic ice cores, which are used for model evaluation. **b** Comparison of measured (red) and modelled (blue) LGM-PD change of *δ*^18^O at 11 Antarctic ice core sites. In addition, simulated *δ*^18^O values are given at four additional sites, where deep ice core drilling projects are ongoing. **c** As **a** but for the simulated LGM-PD change of surface temperatures *T*_surf_. Symbols and text labels mark the position of data from deep Antarctic ice cores. Model values have been calculated for the PD and LGM reference simulations. No correction for glacial seawater change has been applied for measured or modelled LGM-PD *δ*^18^O changes. The period of the last glacial maximum (LGM) is defined as 21 ka before present

**Fig. 3:**
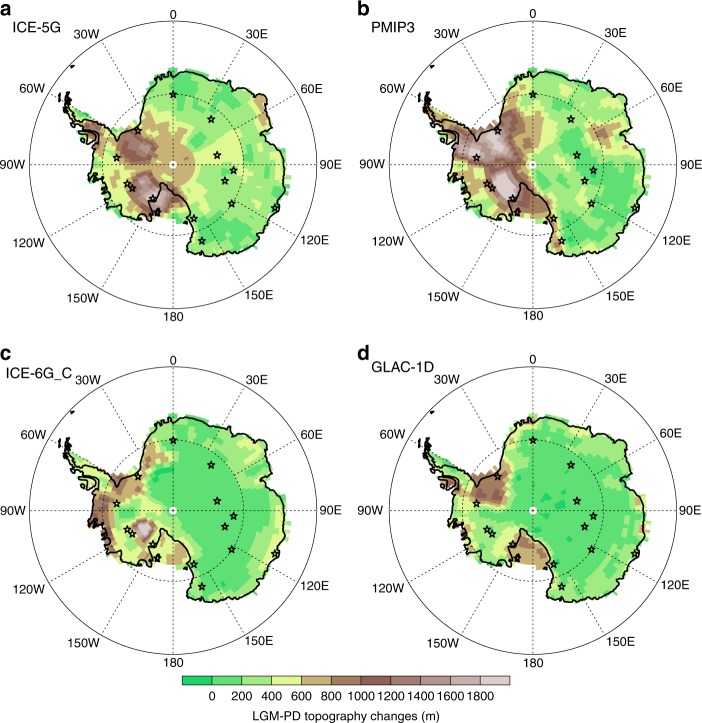
Map of prescribed LGM-PD elevation changes for the Antarctic continent. **a** ICE_5G reconstruction^35^ used for the PMIP2 experiments; **b** PMIP3 reconstruction, a blended product obtained by averaging three different ice sheet reconstructions: ICE-6G v2.0^66^, GLAC-1a^67^, and ANU^68^; **c** ICE-6G_C reconstruction^36,39^; **d** GLAC-1D reconstruction^37,67,69^. The last two reconstructions will be used within the framework of the upcoming PMIP4 simulations^70^. Star symbols mark the position of data from deep Antarctic ice cores

In our LGM reference simulation, model results reveal a strong decrease in surface snow *δ*^18^O, ranging from −2 to −15‰ (Fig. 2a). In most areas of East Antarctica, glacial *δ*^18^O values in snow are lowered by 4–6‰ but decreases reaching −10‰ or more are simulated in West Antarctica east of the Vinson Massif in the Ronne Ice Shelf region and parts of the Marie Byrd Land. For East Antarctica, the simulated LGM *δ*^18^O change is similar at coastal regions and in the East Antarctic plateau, without any clear latitudinal gradient. The magnitude of our simulated glacial *δ*^18^O changes is in general agreement with the available ice core records (Fig. 2b and Table 1), with larger LGM changes at the location of West Antarctic ice cores (modelled values at WDC, Byrd and Siple Dome: −8.4 to −9.3‰) than at East Antarctic ice core locations (modelled values at Vostok, Dome F, EDC, Talos and Taylor Dome: −3.8 to −5.1‰) (Table 1 and Fig. 2b). However, with the prescribed PMIP3 ice sheet, the ECHAM5-wiso model tends to systematically overestimate the LGM changes in West Antarctica, and underestimate those in East Antarctica. The largest model biases occur at Dome B (underestimating *δ*^18^O depletion by 1.4‰) as well as Byrd and WDC (overestimating depletion by 1.7‰ and 2.0‰, respectively). These results are noteworthy as the validity of the PMIP3 Antarctic LGM ice sheet topography has been debated^38^. In West Antarctica, recently measured borehole temperatures at the WDC site indicate a modest LGM ice sheet height change in the range of −300 to +450 m^45^. A modest increase is in better agreement with the ICE-6G_C and GLAC-1D ice sheet reconstructions (Fig. 3).

Two additionally performed sensitivity experiments, which allow a factor separation analysis regarding the influence of PMIP3 ice sheet height versus the other prescribed LGM boundary conditions, have been performed (see Methods). Our results indicate that the stronger LGM cooling and *δ*^18^O anomaly in West Antarctica as compared to the Eastern Plateau is indeed primarily caused by the prescribed LGM changes in ice sheet height (see Supplementary Note 7 for details).

The root mean square error (RMSE) between modelled and measured LGM-PD *δ*^18^O changes for all 11 ice core sites is used as a measure to compare our LGM reference simulation with the additional simulations with alternate ice sheet reconstructions. We find that the PMIP3 ice sheet results in an overall best *δ*^18^O agreement with the ice core data (Table 2). RMSE is 1.1‰ for the PMIP3 ice sheet, 1.5‰ for ICE-5G, 1.9‰ for ICE-6G_C and 2.1‰ for the GLAC-1D reconstruction. Although our LGM reference simulation is outperformed by one of the other model runs for 7 out of the 11 ice core sites, no clear overall regional improvement of neither the older ICE-5G nor the more recent ICE-6G_C or GLAC-1D reconstruction as compared to the PMIP3 one is found.

**Table 2:** Comparison between different prescribed LGM elevation and resulting *δ*^18^O changes

	LGM elevation changes (m)				*ε* (Δ*δ*^18^O) (‰)			
Site	ICE-5G	PMIP3	ICE-6G_C	GLAC-1D	ICE-5G	PMIP3	ICE-6G_C	GLAC-1D
EDML	220	350	210	180	0.7	0.1	1.0	2.2
Dome F	250	340	110	70	0.5	0.8	1.4	1.2
Dome B	290	50	90	50	−1.5	1.4	1.1	0.9
Vostok	300	270	120	40	−2.7	0.9	1.0	−0.4
Law Dome	160	280	390	290	0.9	1.2	−0.2	0.4
EDC	250	280	90	120	−1.0	0.5	0.2	0.1
Taylor Dome	280	660	200	110	1.7	−0.8	2.2	1.1
Talos	140	490	130	220	2.3	0.8	2.6	1.3
Siple Dome	1830	1730	410	670	−1.4	−0.6	4.2	2.8
Byrd	1000	1150	520	250	−1.3	−1.7	2.1	4.7
WDC	650	870	530	240	−1.7	−2.0	0.4	3.0
RMSE (‰)					1.5	1.1	1.9	2.1

The columns list the selected deep-drilling ice core sites, LGM elevation changes at the drilling sites suggested by four ice sheet reconstructions (ICE-5G, PMIP3, ICE-6G_C and GLAC-1D), and deviation *ε* (modelled minus measured) of LGM-PD Δ*δ*^18^O change for the different prescribed ice sheet reconstructions. In the last row, the root mean square error (RMSE) between modelled and measured LGM-PD Δ*δ*^18^O for all 11 ice core sites is given

**Fig. 4:**
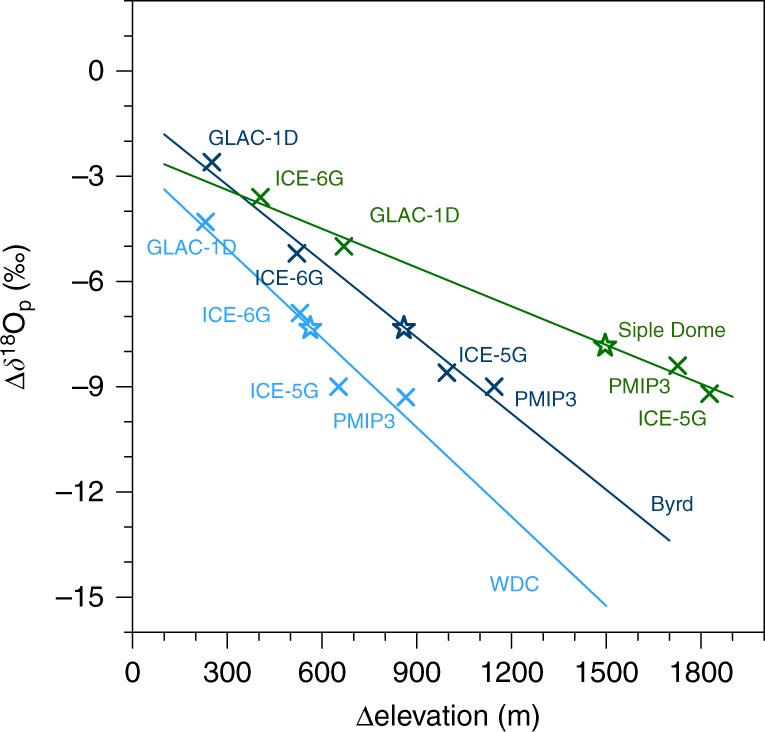
Relation between prescribed LGM-PD ice sheet elevation and simulated *δ*^18^O changes. Modelled *δ*^18^O changes in snow are shown for the locations of the WDC (light blue), Byrd (dark blue), and Siple Dome (green) ice core drilling site. The cross symbols mark the model results of four different prescribed ice sheet reconstructions (ICE-5G, PMIP3, ICE-6G_C, and GLAC-1D). The star symbols mark the measured LGM-PD *δ*^18^O ice core changes

For West Antarctica, our simulations reveal a strong correlation between prescribed LGM ice sheet height and simulated *δ*^18^O changes at the WDC, Byrd and Siple Dome drilling site (Fig. 4). The slopes of the *δ*^18^O–height relations at WDC (−0.8‰/100 m) and Byrd (−0.7‰/100 m) match the simulated PD spatial slope (−0.8‰/100 m), while a 50% lower slope is found for Siple Dome (−0.4‰/100 m). For a 'best guess' of LGM ice sheet height changes, we combine the simulated *δ*^18^O–height relations with the glacial *δ*^18^O changes measured in the three ice cores and assume a potential underestimation of the real *δ*^18^O–height relation by up to 20% in our simulation (like for the observed and simulated PD *δ*^18^O–height slope, see Supplementary Note 7). This approach leads to potential LGM ice sheet height changes of +470 to +560 m at WDC, +720 to +860 m at Byrd and +1250 to +1500 m at Siple Dome, which is lower than the elevation changes suggested by the PMIP3 reconstruction (WDC: +870 m, Byrd: +1150 m and Siple Dome: +1730m). These isotope-based estimates assume that the deviations between modelled and measured glacial *δ*^18^O changes can be solely explained by the prescribed LGM ice sheet changes, with a negligible contribution of the other chosen LGM boundary conditions.

Prescribing glacial isotope changes at the ocean surface based on a fully-coupled isoGCM simulation^46^ instead of a uniform +1‰ with respect to modern observations from the GISS data set^47^ leads to a mean extra depletion of *δ*^18^O in precipitation at the selected Antarctic ice core sites of 0.1‰ only. Thus, the choice of the *δ*^18^O ocean boundary condition appears as noncritical for the following analyses of the validity of the glacial isotope paleothermometer in Antarctica.

If we replace the GLAMAP glacial ocean surface temperatures and sea ice coverage by results from the fully-coupled atmosphere–ocean ECHAM5/MPIOM model set-up^46^, we find a worse agreement between modelled glacial *δ*^18^O changes in precipitation and ice core data. The average absolute model data difference is 1.8‰, about 60% larger as the model bias for our LGM simulation using GLAMAP data (1.1‰), suggesting that cooler and more uniform ocean surface boundary conditions than the ones used in our LGM reference simulation are less plausible (see Supplementary Note 7 for a detailed discussion of these differences).

Overall, we rate our LGM reference simulation with a prescribed PMIP3 ice sheet reconstruction, GLAMAP ocean state and GISS ocean isotope distribution as the best choice for analysing the validity of the isotope paleothermometer in Antarctica (see Supplementary Note 2 for a more detailed evaluation of this LGM climate simulation). But we keep in mind its potential overestimation of isotope depletion in West Antarctica. The simulation is a clear improvement as compared to an older study using the previous model release ECHAM4^2,27^, which can be attributed to three different reasons. First, refined model physics: Various improvements in the representation of the atmospheric water cycle have been implemented in the ECHAM5 model release, such as separate prognostic equations for cloud ice and cloud liquid water, a flux-form semi-Lagrangian transport scheme for all vapour, liquid water and ice in the atmosphere, and a different cloud micro-physical scheme. For Antarctica, we rate the implementation of a separate prognostic equations for cloud ice as most important, but the effect on the simulated *δ*^18^O values in Antarctic presentation are difficult to quantify without substantial changes of the ECHAM5-wiso model code. Second, refined boundary conditions: As discussed above, the PMIP3 ice sheet configuration leads to an improved simulation of glacial *δ*^18^O changes at the selected ice core sites (decrease of RMSE by 0.5‰) as compared to the older ICE-5G data set used in the ECHAM4 study. Third, refined model resolution: In Werner et al.^15^, we have already demonstrated that a higher spatial model resolution is key for an improved simulation of the isotopic composition of precipitation. To quantify this effect for glacial changes in Antarctica, we have repeated both our PI and LGM reference simulation with a coarse T31 spectral resolution (horizontal grid size: 3.8° × 3.8°, 19 vertical levels). For the selected ice core sites, the RMSE increases by only +0.3‰ as compared to our reference simulations (T106 spectral resolution, grid size of 1.1° × 1.1°, 31 vertical levels). The coarser model resolution leads to a lowered glacial ice sheet change and thereby to a better agreement with the ice core data, especially over West Antarctica. However, for both the PD climate and LGM climate, the simulated absolute *δ*^18^O values in precipitation are much more enriched (on average +4.7‰ for PD, +5.5‰ for LGM) for this coarse model resolution and do not fit the ice core data well.

For our LGM reference simulation, glacial surface temperatures in Antarctica are simulated to decrease by 4–20 °C as compared to present-day, changing by 12–20 °C in most regions of West Antarctica, with a weaker cooling, between 4 and 10 °C, for East Antarctica (Fig. 2). As for the *δ*^18^O depletion, the cooling in West Antarctica might be overestimated due to the prescribed high elevation of PMIP3 ice sheet topography, though. Based on a measured borehole temperature profile, Cuffey et al.^45^ estimated at the WDC site a LGM cooling of 11.3 ± 1.8 °C (simulated cooling: 13.8 °C; Table 3). LGM-simulated accumulation rates are all lower than the present ones, as evidenced in ice core estimates^48^. The largest absolute accumulation rate changes are simulated for Law Dome, WDC and Fletcher, and the smallest ones for Vostok and Dome F. For EDC, both reconstruction and simulation results suggest a LGM drying of 50%. For EDML, the simulated reduction is of 37%, while reconstructed values indicate an LGM drying of ~50%. For Berkner and Talos Dome, the simulated LGM accumulation change is up to 20% smaller than the reconstructed one.

**Table 3:** Comparison of modelled spatial and temporal *δ*^18^O-T_surf_ slope and inferred LGM cooling

Site (sector)	Ice Δ*δ*^18^O [‰]	Observed PD slope [‰/°C]	Modelled PD slope [‰/°C]	Modelled temporal slope [‰/°C]	Inferred Δ*T* [°C]	Modelled Δ*T* [°C]
EDML (1)	−6.3	0.79 (0.82)	0.85	0.73 ± 0.10 (33)	−8.9	−9.9
Dome F (2)	−4.9	0.81 (0.80)	0.87	0.68 ± 0.07 (33)	−7.4	−8.0
Dome B (3)	−5.0	0.91 (0.75)	0.88	0.43 ± 0.06 (33)	−8.0	−10.8
Vostok (3)	−4.8	0.91 (0.75)	0.88	0.48 ± 0.13 (33)	−7.7	−11.1
Law Dome (3)	−5.5	0.91	0.88	0.65 ± 0.08 (19)	−7.1^a^	−9.0
EDC (3)	−5.6	0.91 (0.75)	0.88	0.79 ± 0.07 (33)	−8.8	−8.0
Taylor Dome (4)	−4.0	0.70	0.97	0.68 ± 0.15 (31)	−7.1^a^	−8.3
Talos (4)	−5.5	0.70	0.97	0.66 ± 0.18 (33)	−9.3^a^	−9.6
Siple Dome (5)	−7.8	0.88	0.90	1.44 ± 0.47 (33)	−10.0^a^	−5.1
Byrd (5)	−7.3	0.88	0.90	0.65 ± 0.09 (33)	−9.4^a^	−13.7
WDC (5)	−7.3	0.88 (0.70)	0.90	0.70 ± 0.09 (33)	−11.9	−13.8
Dome A (2)			0.87	0.54 ± 0.09 (33)		−7.6
RICE (4)			0.97	1.04 ± 0.35 (27)		−3.9
Fletcher (6)			0.95	0.74 ± 0.18 (33)		−18.4
James Ross (6)			0.95	0.57 ± 0.03 (7)		−14.1
Berkner (6)			0.95	1.09 ± 0.19 (31)		−14.0

The columns list the selected deep-drilling ice core sites, the measured LGM-PD Δ*δ*^18^O, the observed PD slope for the sector within parenthesis the value used for the interpretation of the given ice core, the modelled PD slope in the corresponding sector of each ice core location, and the modelled temporal slope at the site. The number of grid boxes used for calculating the temporal slope is given in brackets. By default, 33 (=11 × 3) grid boxes centred on each drilling location are used. Numbers lower than 33 are caused by the coastal location of some ice cores (ocean grid points are neglected in all calculations). Correction for the prescribed glacial seawater change of 1‰ has been applied to LGM-PD Δ*δ*^18^O values before temporal slope calculation. The second last column reports the LGM-PD temperature change Δ*T* as inferred from the measured LGM-PD Δ*δ*^18^O (also corrected for an assumed glacial seawater *δ*^18^O change of +1‰) and the observed PD slope values used for the interpretation of the different cores. The last column reports the modelled LGM-PD temperature change Δ*T* at the different ice core sites. Further information: Data measured along the Dumont d’Urville- Dome C axis^7^ showing a *δ*^18^O/T_s_ slope of 0.75‰/°C has been used for Dome C^2^, Dome B^42^ and Vostok^17^. A slightly higher value of 0.85‰/°C is observed in the Dome Fuji sector but an average with the Vostok sector of 0.80‰/°C is taken into account by Watanabe et al.^33^ as this study focuses on the comparison between the Dome F and Vostok temperature records. A similar value of 0.82‰/°C is reported for Dronning Maud Land area and used for interpreting the EDML isotopic profile^5^. For the WAIS divide core, the temperature interpretation also uses isotopic profiles but calibrated using a measured borehole temperature profile, the estimate of the LGM cooling of 11.3 ± 1.8 °C^[45]^ would be consistent with the use of a slope close from 0.7‰/°C

^a^Inferred Δ*T* values have been calculated using the observed PD slope in the related sector

### The isotopic paleothermometer for LGM changes in Antarctica

Initially, simple Rayleigh-type isotopic models have been applied to justify the use of the observed present-day *δ*^18^O-*T*_surf_ spatial slope (referred hereafter as slope *m* and expressed in ‰/°C) as a surrogate of the temporal slope to interpret ice core isotopic records. Such a model correctly accounts for the observed present-day linear relationship between the isotopic composition of snow and the temperature of the site^8^ and predicts similar slopes for present-day and LGM conditions. The same approach of comparing observed, present-day and LGM slopes can be followed from our isoGCM simulations. First, the observed linear relation between present-day values of *δ*^18^O in snow and *T*_surf_ is well simulated by ECHAM5-wiso (see Supplementary Note 3 for details). Despite the modelled warm, wet and enriched biases, the simulated spatial slope (*m* = 0.77 ± 0.01‰/°C) is very close to the observed relationship (*m* = 0.79 ± 0.01‰/°C). The observed and simulated spatial slopes are also very close to Rayleigh distillation lines in both West and East Antarctica, when they are considered separately (West Antarctica: observed: *m* = 0.84 ± 0.03‰/°C, modelled: *m* = 0.82 ± 0.03‰/°C; East Antarctica: observed: *m* = 0.85 ± 0.01‰/°C, modelled: *m* = 0.78 ± 0.01‰/°C). Simulated LGM spatial slopes for both West and East Antarctica are similar to the simulated present-day ones. Despite the much stronger simulated LGM cooling in West Antarctica, the modelled *δ*^18^O-*T*_surf_ slope in this region (*m* = 0.88 ± 0.01‰/°C) follows the same distillation line as simulated in East Antarctica (*m* = 0.85 ± 0.01‰/°C).

**Fig. 5:**
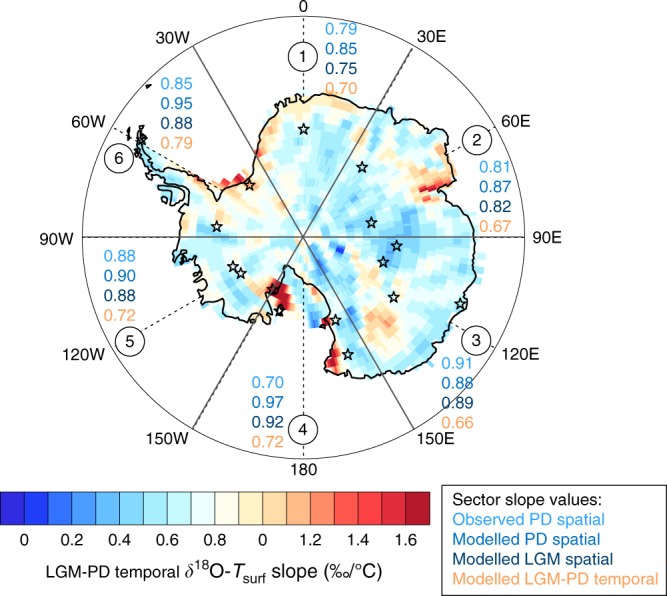
Map of simulated LGM-PD temporal *δ*^18^O-*T*_surf_-slopes, calculated for the PD and LGM reference simulations. Symbols (stars) mark the position of data from deep Antarctic ice cores, which are used for model evaluation. The numbers in circles indicate the defined six different sectors of Antarctica, for which regional analyses are applied. The coloured row of numbers in each sector states the following *δ*^18^O-*T*_surf_-slopes: observed PD spatial (light blue), modelled PD spatial (blue), modelled LGM spatial (dark blue), and modelled LGM-PD temporal slope (orange). All slopes are given in ‰/°C

Antarctic ice core data are generally interpreted using the present-day spatial *T*_surf_-*δ* (*δ*^18^O or δD) slope observed over a large area around the ice core site. In this line, we have divided Antarctica in six large sectors (Fig. 5) and compare, for each of these sectors, the observed present-day spatial slope, the simulated present-day and LGM spatial slopes, and finally the temporal slope simulated in this sector. This temporal slope is calculated as the modelled LGM-PD Δ*δ*^18^O precipitation anomaly divided by the modelled LGM-PD Δ*δT*_surf_ anomaly. Simulated LGM *δ*^18^O anomalies are corrected for the glacial enrichment of ^18^O in seawater (+1‰) prior to the calculations of the temporal slopes^27^. Over the six sectors, the temporal slope is slightly—but consistently—lower than the present-day observed and modelled spatial slope, by 17–26%. Using the isotopic paleothermometer based on PD observations, therefore, implies an underestimation of LGM-PD temperature change by this amount. Daily temperatures weighted by precipitation amount should in principle lead to a better fit between annual mean isotope and temperature changes, as the link between these two quantities is only expected to hold during precipitation events. When calculating the slopes using precipitation-weighted temperature, we indeed obtain very similar slope values between modelled PD slopes and temporal slopes, with deviations lower than 8% for five of the six Antarctic sectors (see Supplementary Note 4 for details). These results support the general idea that isotope–temperature relations should be used for a reconstruction of precipitation-weighted mean temperatures, rather than annual mean temperatures (e.g., refs. ^49–51^). This holds also true for spatial slopes, which should be ideally based on precipitation-weighted temperatures. However, a precipitation weighting cannot be applied when using observations, only.

Previous studies have also investigated the possibility of using seasonal changes in *δ*^18^O and temperatures for calibrating the Antarctic isotopic thermometer. For our reference simulation, we find seasonal slopes which are substantially lower than the simulated spatial slopes both for present-day and LGM conditions. These lower slopes are in agreement with observations and would imply to an unrealistic strong LGM cooling in Antarctica (see Supplementary Note 5 for details).

Recent measurements of the isotopic composition of surface air above both the Greenland and Antarctic ice sheet have indicated a post-depositional isotope exchange between vapour and snow^52–54^. Our model results suggest that such exchange might not alter the investigated *δ*^18^O-*T*_surf_ slopes in a considerable manner (see Supplementary Note 6 for details).

For the different deep ice core sites in Antarctica, the similarity between spatial and temporal slopes should be tested at a more local scale to validate the isotopic paleothermometer. For example, this can be done by calculating LGM-PD temperature changes using the observed LGM-PD isotopic change (seawater corrected) and the observed present-day spatial slope. The results are listed in the second last column of Table 3 for the 11 sites with measured LGM isotopic data. However, to test the validity of the isotopic paleothermometer we focus on the sites Vostok, Dome F, Dome B, EDC, EDML and WDC, for which observed present-day spatial slopes on a local scale have been reported. Again, the use of the local isotopic paleothermometer leads to an underestimation of the modelled LGM cooling by ~15%, with however a relatively large spatial heterogeneity.

Our simulations support the validity of the modern analogue hypothesis for the Vostok and EDC record based on previous findings derived from ECHAM4-wiso simulations^2,27^ with however a slight systematic underestimation, by about 15%, of modelled LGM cooling. Our findings thus expand the conclusions of a previous study^55^, suggesting that the isotope-based approach (using PD spatial slopes) slightly underestimates glacial temperature changes on the East Antarctic Plateau, to the whole Antarctic continent despite the different characteristics of the various isotopic records in West and East Antarctica during the last glacial maximum and deglaciation^32^. These findings hold also true despite the overestimation of the glacial West Antarctic ice sheet changes in the PMIP3 reconstruction, as temperature and *δ*^18^O changes are both strongly linked to the prescribed height of the glacial ice sheet. It is noteworthy that such match of the spatial and temporal slopes may not hold for a shift to a warmer climate in Antarctica^30^, and neither for glacial and last deglaciation *δ*^18^O-*T*_surf_ slopes in Greenland^20,56–58^.

We conclude that the ECHAM5-wiso simulation results are in good agreement with observed present-day Antarctic surface temperatures and *δ*^18^O values in surface snow. As for many other AGCMs^40,41^, a warm model bias still exists over the Antarctic continent, despite the chosen high-spatial model resolution. Simulated LGM *δ*^18^O anomalies are also in good agreement with ice core data. The observed stronger depletion of water isotopes in West Antarctic deep ice cores as compared to East Antarctic ones can be related to a stronger LGM cooling of the former. This larger cooling and isotopic depletion is attributed to the prescribed glacial ice sheet elevation changes. Our model results with prescribed different LGM ice sheet reconstructions of Antarctica indicate an overall good match of the modelled glacial *δ*^18^O changes for the PMIP3 reconstruction. The suggested West Antarctic elevation changes seem to be too large by 30–50% at the Byrd and WDC drilling site, though.

Observed and modelled spatial *δ*^18^O-*T*_surf_ slopes are in good to very good agreement on a large geographical scale, i.e., for East and West Antarctica, as well as on a smaller sector scale for the present-day climate. It should be noted though that the sector scale agreement might depend on the choice of the spatial region^29^. For the LGM, modelled spatial slopes are almost identical to the present-day ones. Analyses of the different ECHAM5-wiso simulations indicate that the LGM present-day temporal *δ*^18^O-*T*_surf_ slope is slightly lower as compared to the mean present-day spatial value for all regions of Antarctica. Thus, in accordance with previous findings for a few selected East Antarctic sites, the classic δ^18^O paleothermometer approach is supported by these new ECHAM5-wiso model results for the whole Antarctic continent. Applying the mean spatial *δ*^18^O-T slope for calculating last glacial maximum temperature changes from Antarctic ice core records might lead to a systematic underestimation, on average of around 15%, only.

In this study, we do not only compare our model results to already published Antarctica ice core data, but also report model findings for some of the more recent, ongoing and future drill sites. Further deep-drilling projects are already in progress, e.g., the search for finding 1.5 million years old ice in Antarctica^59^. Our simulation results might also help to constrain estimates of past temperature changes from some of these upcoming new ice core *δ*^18^O data.

## Methods

### Model description

ECHAM5-wiso is an atmospheric general circulation model that also explicitly allows the simulation of the three isotopic water species H_2_^16^O, H_2_^18^O and HDO^15^. This model has its origin in the fifth version of the atmospheric general circulation model ECHAM5^60^ and is enhanced by including a water isotope module in the model’s hydrological cycle, following the work of Joussaume et al.^9^, Jouzel et al.^10^ and Hoffmann et al.^11^. The isotope module computes changes of the composition of different water masses within the entire hydrological cycle, including evaporation from the ocean, cloud condensation, precipitation, surface water reservoirs and river runoff^15^.

### Simulation set-up

Two different simulations were performed for the present-day climate. For our PD reference simulation, orbital parameters and GHG concentrations are set to modern values. For the prescribed ocean surface state, we used AMIP-style boundary conditions^61^, including prescribed monthly climatological sea surface temperatures (SST) and sea ice cover for the period 1979–1999. The isotopic composition of surface seawater is prescribed using the global gridded data set of LeGrande and Schmidt^47^. As no equivalent data set of the *δ*D composition of seawater exists, the deuterium isotopic composition of the seawater in any grid cell has been set equal to the related *δ*^18^O composition, multiplied by a factor of 8, in accordance with the observed relation for meteoric water on a global scale^62^.

In a second PD simulation, we use the same boundary conditions as for the reference one, but vary the prescribed isotopic composition of surface seawater by using results obtained with a fully-coupled ocean–atmosphere GCM including isotope diagnostics^46^. For this simulation, the modelled pattern of *δ*^18^O in seawater is in agreement with the data set of LeGrande and Schmidt^47^ on a global scale, but regional differences of up to ±0.4‰ exist.

For the LGM climate, six different simulations have been performed. For all simulations, the set-up of orbital parameters, greenhouse gas concentrations (GHG), and land-sea distribution follows the guidelines of the Paleoclimate Modelling Intercomparison Project Phase III (PMIP3, https://pmip3.lsce.ipsl.fr). Prescribed glacial climatological monthly sea ice coverage and SST changes are based on the GLAMAP data set^63^, which combined the global CLIMAP^64^ ocean state reconstruction with more recent LGM temperature and sea ice estimates of the Atlantic^65^. A uniform glacial enrichment of sea surface water and sea ice of +1‰ (*δ*^18^O) and +8‰ (*δ*D) on top of the present-day isotopic composition of surface seawater has been applied.

For our LGM reference simulation, the ice sheet extension and height change also follow the guidelines of PMIP3. Proposed LGM ice sheet changes are a blended product obtained by averaging three different ice sheets reconstructions: ICE-6G v2.0^66^, GLAC-1a^67^ and ANU^68^. Key regional changes include an increase of ice sheet height of up to 2 km in major parts of West Antarctica as well as a local increase in ice sheet height of about 1 km near Prince Charles Mountains, Mac Robertson Land. Most parts of the East Antarctic interior remain unchanged or include only minor glacial height changes below 400 m.

In a second LGM simulation, we have prescribed the ice sheet reconstruction ICE-5G^35^ used for the previous PMIP Phase II experiments (PMIPII, https://pmip2.lsce.ipsl.fr). In ICE-5G, a less (stronger) increase of LGM ice sheet height in West (East) Antarctica as compared to the PMIP3 reconstruction was suggested^38^. In two further simulations, we prescribe two more recent glacial ice sheet reconstructions, ICE-6G_C^36,39^ and GLAC-1D^37,67,69^. These two reconstructions will be used within the framework of the upcoming PMIP4 simulations^70^. Both reconstructions suggest the least ice sheet height and extension changes in West Antarctica, and also slighlty lower changes of the glacial East ice sheet height as compared to the PMIP3 reconstruction.

In a fifth LGM simulation, we vary the prescribed isotopic composition of surface seawater by using results obtained with a fully-coupled ocean–atmosphere isoGCM^46^. Together with the corresponding PD simulation it allows us to estimate the influence of different prescribed *δ*^18^O values in seawater on the simulated glacial *δ*^18^O changes in Antarctic precipitation.

For a sixth LGM simulation, we have replaced the GLAMAP-based glacial sea ice coverage and SST changes by values derived from an LGM climate study with the fully-coupled ocean–atmosphere model set-up ECHAM5/MPIOM-wiso^46,71^. This simulation is used to explore the dependence of simulated glacial *δ*^18^O changes in Antarctic precipitation on the prescribed glacial ocean surface state.

For a factor separation analysis of ice sheet height versus other glacial boundary conditions on the simulated isotope signal in Antarctica, we have performed two further sensitivity experiments. In the first sensitivity experiment, we use present-day boundary conditions but increase the Antarctic ice sheet height to PMIP3 LGM values. In the second one, we use all LGM boundary conditions as in our LGM reference simulation but decrease the Antarctic ice sheet height to present-day values. These two experiments, combined with the PD and LGM reference simulations, allow us to separate the effect of glacial Antarctic PMIP3 ice sheet height from other glacial boundary conditions (orbital parameters, greenhouse gases and ocean state).

Former tests with ECHAM5-wiso had evidenced the sensitivity of model performance for Antarctic stable water isotope distribution to model resolution, and an overall consistency with observations at T106, probably because this resolution allows a reasonable representation of moisture transport within storms^15^. All ECHAM5-wiso simulations in this study have therefore been run using the high-spatial T106 model resolution (approx. horizontal grid size: 1.1° × 1.1°) with 31 vertical model levels between surface and 10 hPa. Reported model results are mean values of the last 10 simulation years, with a total simulation period of 22 years for the present-day and LGM reference simulation, and 12 years for all other experiments, respectively. If not stated otherwise, all model mean *δ* values in this study are calculated as amount-weighted averages.

## Electronic supplementary material

Supplementary Information

Reviewers' comments

## Data Availability

The model data that support the findings of this study are available from the corresponding author on reasonable request.
