# Supplementary material for: Reconciling glacial Antarctic water stable isotopes with ice sheet topography and the isotopic paleothermometer
Source: Nat Commun. 2018 Aug 30;9:3537. doi: 10.1038/s41467-018-05430-y (PMC6117368; doi:10.1038/s41467-018-05430-y)
Supplement: Supplementary file 1 — Supplementary Information [file 41467_2018_5430_MOESM1_ESM.pdf]

Supplementary Note 1– Model evaluation for present-day climate

For present-day, numerous annual mean Antarctic surface temperatures, accumulation rates and isotopic values of Antarctic snowfall are documented[1-3] and Masson-Delmotte et al.[4] compiled all observational Antarctic data sets, available at that time. For net accumulation over Antarctica, Favier et al.[5] recently presented an updated, quality-controlled database. In these compilations, reported surface temperatures (T_{surf}) are a blend of annual mean surface temperature inferred from firn temperature measurements, as well as averages of 2m air temperatures, for different averaging periods and different stations. Net accumulation rates may include measurements of solid precipitation, liquid precipitation, erosion by wind, sublimation and runoff, respectively, but again the available data is a blend of different measurements, which might not always include all these terms in an equal manner. This heterogeneity in the available present-day observational data has to be kept in mind when comparing these data to the ECHAM5-wiso modelling results.

Within our study, we found that 123 out of 1279 records of the Masson et al.[4] database, all from one dataset from the area near Kohnen station, contained wrong accumulation values due to a misunderstanding of the units in the initial publication. We exclude these data points from our analyses and select from the remaining database only those records, which contain values of both T_{surf}, and $\delta^{18}O$. This filtering results in a subset of 552 entries (named MD08 data set, hereafter). Within MD08, only 180 data points also contain accumulation data. For expanding the evaluation of modelled Antarctic accumulation rates, we also compare our model values to the set of 3209 records of quality-controlled net accumulation rate reported by Favier et al.[5].

Simulated annual mean Antarctic surface temperatures range from -10°C to -55°C, with warmest temperatures in coastal areas as well as the Antarctic Peninsula and coldest

temperatures at high elevations and most inland areas, and therefore on the East Antarctic ice sheet (Fig. 1a). The modelled mean temperature of West Antarctica (-30.5°C) is 10.6°C higher than for East Antarctica (-41.1°C). Both temperature distribution pattern and simulated absolute values are in good agreement with the observed temperature values in MD08 (Fig. 1a, 1b). However, the ECHAM5-wiso model has an overall warm bias, which grows from zero to 5°C in the temperature range of -20°C to -35°C. Such a warm bias over Antarctica is common to many isotope-enabled AGCMs[6,7] and frequent in GCM simulations[8,9]. It might be caused by poor representation of the polar atmospheric boundary layer and related atmospheric inversion temperatures in GCMs[10]. A linear correlation analysis between observed and simulated surface temperatures results in a slope of m = 0.85±0.01 and a correlation coefficient of r = 0.94. The root-mean-square error (RMSE) of the model results is 4.6°C.

Accumulation estimates can be compared with the simulated precipitation minus evaporation (P-E) model output, as a surrogate for accumulation. Re-location of snow by wind drift, which might strongly impact the observed estimate of accumulation at a specific location on the ice sheet[5], is not parameterized in ECHAM5-wiso. All accumulation values are expressed in water equivalent rates. The present-day simulated accumulation ranges between 2cm/yr and 80 cm/yr. The distribution of the simulated accumulation is also similar to the spatial structure of temperature and $\delta^{18}O$. Highest accumulation rates are simulated on the Antarctic Peninsula as well as in the coastal regions between 60°W-150°W and 90°E-150°E at elevations below 1000m (Fig. 1e). A very dry region with mean accumulation rates less than 5cm/yr is simulated in the East Antarctic plateau, with lowest values in the vicinity of Dome C. We stress the fact that the cloud and precipitation scheme of ECHAM5 had not included any specific adjustment for the specific forms of condensation and precipitation processes in Antarctica[11,12]. The overall good agreement between simulated and estimated accumulation fluxes from the MD08 data set is therefore remarkable (m = 0.95±0.06, r = 0.69,

RMSE = 12.1 cm/yr). Nevertheless, the model has a general wet bias, which is consistent with its warm temperature bias. Most of the model-data mismatch occurs in coastal areas (accumulation rates > 20cm/year), where ice core based reconstructions of past climates might be complicated by high spatial accumulation variability[13]. Focusing on the areas where most deep ice cores are located, with accumulation fluxes less than 10cm/yr, estimated and simulated accumulation rates are in even better agreement (Fig. 1f). For the much larger recent Favier et al.[5] data set of 3209 records of net accumulation, we calculate a similar good agreement between simulated and estimated accumulation fluxes (m = 0.93±0.02, r = 0.78, RMSE = 12.2 cm/yr). Compared to ERA-40 data, the simulated mean precipitation of ECHAM5, used in a fully-coupled AOGCM setup, is slightly higher[14] but in a comparison with other models participating in the CMIP3 intercomparison study, ECHAM5 performed relatively well for simulating the precipitation over the Antarctic continent[15].

For isotope data, we compare model outputs for snow $\delta^{18}O$ with data obtained from surface snow or ice core records. In ECHAM5, surface snow $\delta^{18}O$ is simulated for a 1-layer bucket scheme, which integrates modelled precipitation amount, diffusion and rime formation. Simulated $\delta^{18}O$ values in surface snow range between -18‰ and -55‰, and their distribution mimics that of the simulated temperature (Fig. 1c). Highest (lowest) $\delta^{18}O$ values are simulated in coastal areas and the Antarctic Peninsula (East Antarctic plateau). At the sample locations of the MD08 data set, ECHAM5-wiso $\delta^{18}O$ values are, on average, 5‰ less depleted than the measured isotope values; this is consistent with the modelled warm bias and insufficient distillation. Modelled and measured $\delta^{18}O$ values are as strongly correlated as for temperature (m = 0.82±0.01; r = 0.92; RMSE = 5.3‰; Fig 1d). A similar bias, but for some models with a larger offset up to +10‰, has been reported for the isotope-enabled versions of LDMZ4, CAM2, ECHAM4, GISS, and MIROC model[16], in correspondence with the warm Antarctic temperature bias also depicted by these models.

Supplementary Note 2 – Model evaluation for LGM climate

An evaluation of the ECHAM5-wiso results is first carried out as a comparison to a set of PMIP3 simulated LGM temperature changes[17]. As compared to the average of PMIP3-participating models[18], the ECHAM5-wiso results of our reference simulations indicate an up to 5°C stronger cooling in West Antarctica, and a stronger cooling between 2°-5°C in East Antarctica. For individual model simulations, the ECHAM5-wiso results are most comparable to the LGM cooling found for the COSMOS-ASO, the IPSL-CM5A-LR, the MPI-ESM-P, and the MRI-CGCM3 coupled simulations. Simulated accumulation and precipitation changes in ECHAM5-wiso are similar to the average results of the PMIP3 models. Largest precipitation changes in the range of 0.25 - 0.5 mm/day occur over parts of West Antarctica (mainly Ellsworth Land) while for most other regions of the Antarctic ice sheets absolute precipitation changes are lower than 0.25mm/day (see synthesis maps at http://pmip3.lsce.ipsl.fr for details). For this model-model comparison it should be kept in mind that all PMIP3 model results stem from fully coupled ocean-atmosphere GCM setups. In contrast, the ECHAM5-wiso temperature and accumulation results may depend on the prescribed glacial ocean state, which is based on the GLAMAP data set (see also discussion in Supplementary Note 7).

So far, only two estimates of glacial-interglacial temperature changes have been inferred from the inversion of Antarctic borehole temperature profiles, using different methodologies. For Vostok, Salamatin et al.[19] have analysed a borehole temperature profile and report a glacial-interglacial temperature change over central Antarctica of 15°C at the surface and about 10°C at the atmospheric inversion, where most precipitation is formed. For the LGM, they estimate a cooling of 11.7°C at Vostok. Our simulation results reveal a cooling of 11.1°C at Vostok, in close agreement with this former estimate. Applying a slightly different approach by combining borehole temperatures, δD measurements and the nitrogen isotopic composition of

trapped gas ($\delta^{15}N$), Cuffey et al.[20] suggest a cooling of 11.3±1.8°C at the WDC drill site for the LGM compared to the late Holocene (present to 3 ka B.P.). The glacial cooling simulated with our ECHAM5-wiso PD and LGM reference simulation is -13.8°C, about 20% larger than this estimate. However, Cuffey et al.[20] consider in their analyses a rather modest LGM-PD change of ice thickness height (-300m to +450m) at the WDC drill site, while an LGM increase of +870m is assumed in our LGM reference simulation with a prescribed PMIP3 ice sheet reconstruction. As discussed in the main text, for WDC an LGM height increase of approx. 560m might be more in line with the LGM $\delta^{18}O$ depletion found in the WAIS ice core. Capron et al.[21] have reconstructed LGM temperatures and accumulation rates at five ice core sites (EDML, EDC, Talos, Berkner Island, and James Ross Island) based on the water isotopic record of each ice core. Calculations were done with locally varying parameters describing the temperature-isotope and accumulation-isotope relation at each site. We compare the published data at the ice core sites with the corresponding simulated ECHAM5-wiso values. Despite the warm bias of the ECHAM5-wiso model for modern climate conditions, simulated LGM-present cooling is in good agreement (deviations <1.5°C) with the reconstructed values at EDC, EDML and Talos. For Berkner Island, model results indicate a LGM cooling of only -14°C, which is 5°C less than the reconstructed value of -19°C. Interestingly, Berker Island is also the only location where the ECHAM5-wiso model does not show a warm temperature bias for the present-day climate, but a cold offset of -2.4°C. The reason for this cold bias remains open, as the altitude of this location in ECHAM5-wiso (340m a.s.l.) is lower than the real altitude (890m a.s.l.). As for the other three drill sites, one would rather expect a warm modelled temperature bias caused by such an underestimation of the altitude. For the LGM, simulated accumulation rates are all lower than the present ones. In agreement with the findings of Capron et al.[21], largest absolute decreases of accumulation occur at Berkner Island, and smallest absolute changes at EDML and EDC. Both

reconstruction and simulation results suggest a drying of 50% at the EDC drill site. However, while reconstructed values also indicate an LGM drying of approx. 50% at the EDML drill site, the simulated LGM accumulation changes by 37% as compared to the modern one, only. For Berkner and Talos Dome, the simulated LGM accumulation change is approx. 20% smaller than the reconstructed one. While these model-data differences in glacial temperature and accumulation change might be caused ECHAM5-wiso model deficits, we would like to emphasise again that the reconstructed LGM values by Capron et al.[21] are based on the water isotopic record of each ice core. For the conversion of temporal isotope changes into temperature variations, local (modern) spatial temperature-isotope slopes at the different drill sites have been used.

Jasechko et al.[22] compared late-glacial to late-Holocene precipitation $\delta^{18}O$ changes derived from groundwater, speleothem and ice core data with simulation results of five isotope-enabled GCM: CAM3iso[23], ECHAM5-wiso[24], GISSE2-R[25], IsoGSM[26], and LMDZ4[7]. Simulated results of $\delta^{18}O$ were derived for each GCM from a modern and a LGM simulation. The modern climate simulation was based on either present-day (ECHAM5-wiso, IsoGSM) or pre-industrial (CAM3iso, GISSE2-R, LMDZ4) boundary conditions. The applied LGM boundary conditions (e.g., sea surface temperatures, sea ice cover) varied between the different models (see Table S1 in ref. 22 for details). For Antarctica, this model inter-comparison revealed that ECHAM5-wiso is the only model that simulated a glacial decrease of $\delta^{18}O$ in precipitation over the whole Antarctic ice sheet. CAM3iso results show positive LGM-present $\delta^{18}O$ anomalies over East Antarctica, while both GISSE2-R and LDMZ4 simulate positive $\delta^{18}O$ anomalies over some coastal regions of Antarctica and in Victoria Land at the eastern coast of the Ross Sea (Fig. 5 in ref. 22). Antarctic $\delta^{18}O$ values of the IsoGSM model could not be evaluated due to data unavailability. Although the ECHAM5-wiso results over Antarctica appear in better agreement with ice core records than the other GCM results,

this conclusion has to be taken with caution as it might be partly caused by the different boundary conditions applied for the five GCMs in this study.

Supplementary Note 3 – Spatial relation between temperature, accumulation and δ¹⁸O in snow

The observed strong relation between present-day values of $\delta^{18}O$ in snow and T_{surf} (Supplementary Figure 1a) is well simulated by ECHAM5-wiso (Supplementary Figure 1b). Despite the modelled warm, wet and enriched biases, the simulated spatial slope calculated from model outputs at the grid points corresponding to all MD08 locations (m = 0.77±0.01‰/°C, r = 0.95, number of data points N = 540) is very close to the observed relationship (m = 0.79±0.01‰/°C, r = 0.96, N = 551). If we include the simulated values of all Antarctic grid points instead of a subset at the MD08 locations, only, the slope just slightly increases by about 15% (m = 0.90±0.002‰/°C, r = 0.97, N = 5206). This indicates that the T_{Surf}-$\delta^{18}O$ relationship derived from the MD08 locations can be used for whole Antarctica. The observed and simulated spatial slopes are also very close to Rayleigh distillation lines in both West and East Antarctica, when they are considered separately (West Antarctica: observed: m = 0.84±0.03‰/°C, N =120; modelled: m = 0.82±0.03‰/°C, N = 112; East Antarctica: observed: m = 0.85±0.01‰/°C, N = 431; modelled: m = 0.78±0.01‰/°C, N = 428; Supplementary Figure 1c). For the LGM simulation, we find for both West and East Antarctica simulated spatial T_{Surf}-$\delta^{18}O$ relationships, which are in close agreement with the modelled present-day values (Supplementary Figure 1d). Despite the much stronger LGM cooling of the West Antarctic ice sheet, the related $\delta^{18}O$ changes (m = 0.88±0.005‰/°C, N = 2178) follow the same distillation line as the East Antarctic values (m = 0.85±0.004‰/°C, N

= 3028). Supplementary Figure 2a shows that the present-day $\delta^{18}O$ in snow and T_{surf} are also very well linearly correlated in each of the six sectors defined in Fig. 5 with a present-day slope varying from 0.85‰/°C to 0.97‰/°C, in good agreement with the slope estimated from the MD08 dataset. As illustrated in Supplementary Table 1, the present-day and LGM slope (Supplementary Figure 2b) are quite similar in each of these sectors.

Besides the close link between surface temperatures and $\delta^{18}O$ in snow, we also analyse the simulated spatial relationship between $\delta^{18}O$ and accumulation (Supplementary Figure 3). Based on theoretical calculations using the Clausius-Clapeyron relation of temperature vs. water holding capacity of the atmosphere as well as observational data from Antarctica, an exponential relation between isotopes in surface snow samples and accumulation rates has been reported[21,27]. At the temporal scale, such relationship has been used for the construction of ice core chronologies[28,29]. For the present-day observational MD08 data set used in this study, we observe an exponential $\delta^{18}O$-accumulation-relation (Supplementary Figure 3a) with a slope $b = 0.076 \pm 0.003‰^{-1}$ for $\delta^{18}O$ as a linear function of log (accumulation) and a strong correlation between $\delta^{18}O$ and accumulation (r = 0.86, N=206). A similar exponential correlation is observed for the simulated present-day values of $\delta^{18}O$ in snow and accumulation (Supplementary Figure 3b). The modelled slope ($b = 0.10 \pm 0.003‰^{-1}$) is approx. 30% higher than the observed one, though, with a comparable strong correlation coefficient (r = 0.92, N=198). For the LGM, this relationship stays constant, despite lowered glacial values of both $\delta^{18}O$ and accumulation. These results indicate that the simulated accumulation rates in ECHAM5-wiso above Antarctica indeed follow an exponential relation with respect to spatial isotope and surface temperature variations. However, the modelled amount of accumulation decreases somewhat stronger with cooler temperatures (as indicated by lower isotope $\delta^{18}O$ values) than observed for present-day.

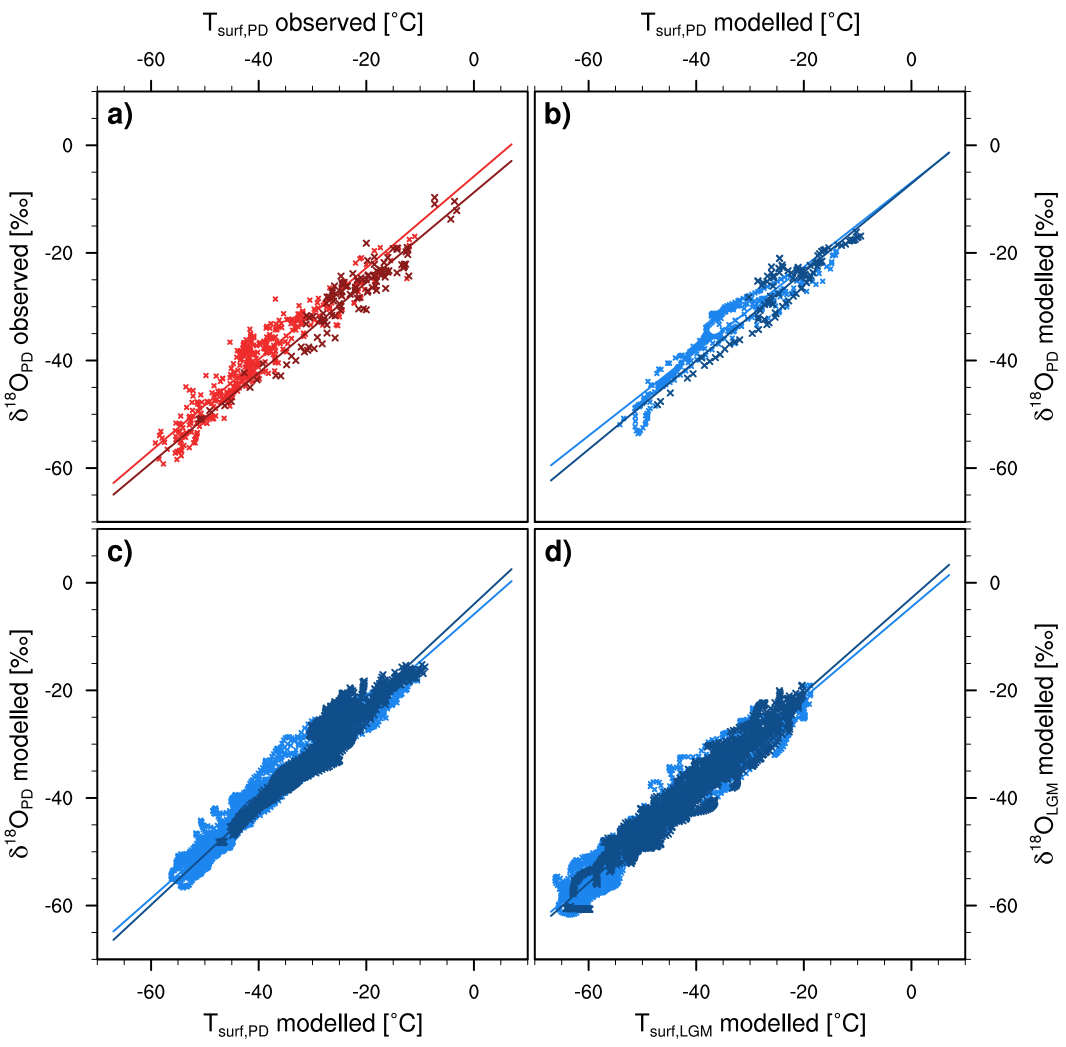

Supplementary Figure 1. Present-day spatial relation between $\delta^{18}O$ in snow and Antarctic surface temperatures. a) Observed present-day spatial $\delta^{18}O$-T_{surf}-relation for the West (dark red) and East (light red) Antarctic ice sheet. Data have been compiled by Masson-Delmotte et al[4]. b) As a) but as simulated by ECHAM5-wiso at the locations of observations for mean present-day climate conditions (PD reference simulation; dark blue: West Antarctica; light blue: East Antarctica). c) Simulated present-day spatial $\delta^{18}O$-T_{surf}-relation for all grid points of the West (dark blue) and East (light blue) Antarctic ice sheet. d) as c) but for the simulated LGM spatial $\delta^{18}O$-T_{surf}-relation of the LGM reference simulation.

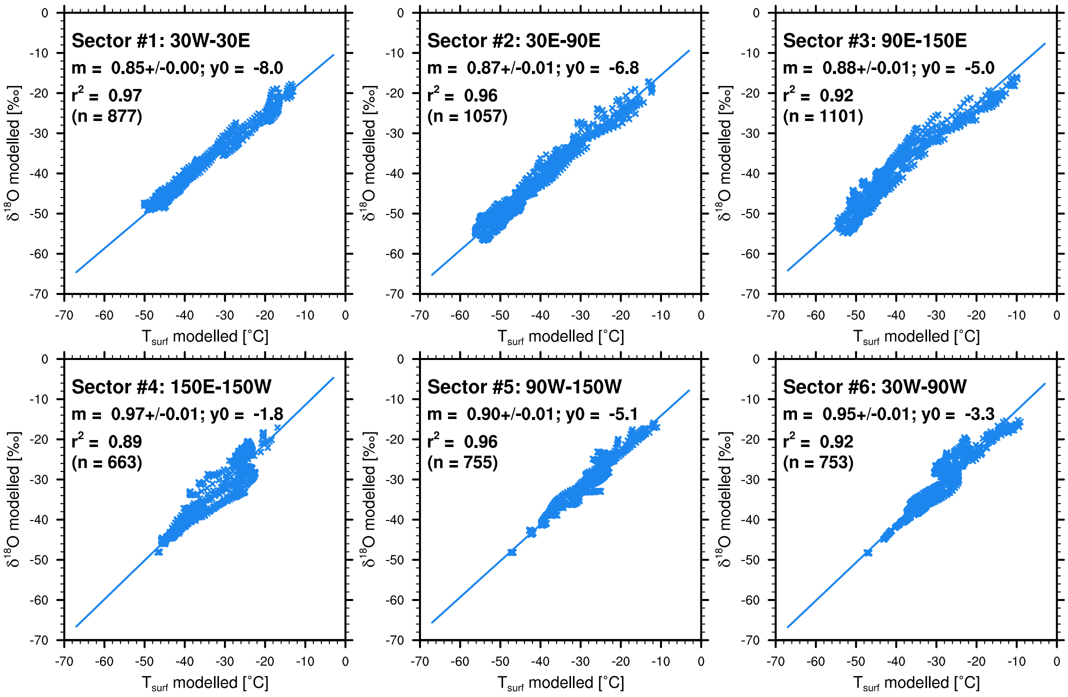

Supplementary Figure 2a. Simulated present-day spatial $\delta^{18}O$-T_{surf}-relation for the sectors defined in Figure 5.

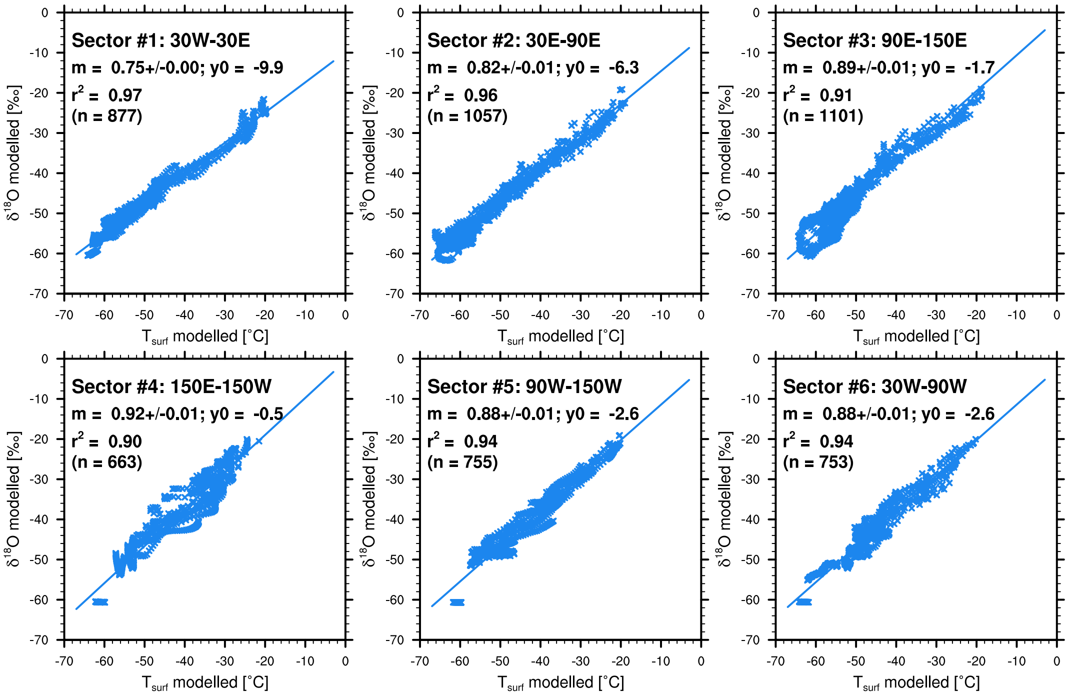

Supplementary Figure 2b. Simulated LGM spatial $\delta^{18}O$-T_{surf}-relation for the sectors defined in Figure 5.

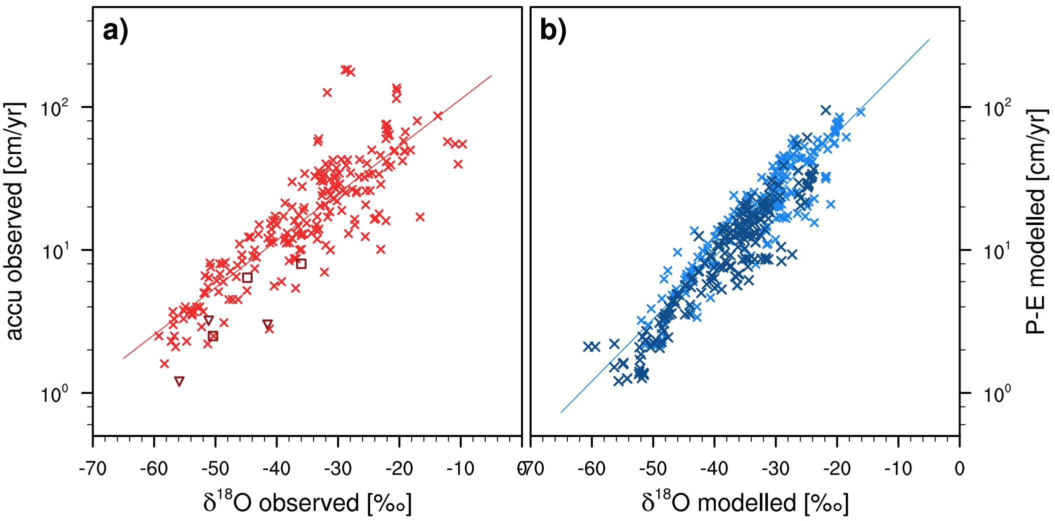

Supplementary Figure 3. Present-day spatial relation between δ^{18}O in snow and Antarctic snow accumulation. a) Observed present-day spatial δ^{18}O-accumulation-relation for Antarctica. Data have been compiled by Masson-Delmotte et al[4]. Ice core values from EDML, EDC, and Talos Dome (ref. 21, 29) are marked by dark red symbols (present-day: squares; LGM: triangles). b) As a) but as simulated by ECHAM5-wiso at the locations of observations for mean present-day (light blue) and LGM (dark blue) climate conditions. Please note the logarithmic y-axis scaling in both plots. Model values have been calculated for the PD and LGM reference simulation.

Sector	Observed PD slope [‰/°C]	Modelled PD slope [‰/°C]	Modelled LGM slope [‰/°C]	Modelled Temporal Slope [‰/°C]
1	0.79 (N=38)	0.85 (0.77*)	0.75 (0.72*)	0.70 (0.76*)
2	0.81 (N=22)	0.87 (0.85*)	0.82 (0.78*)	0.67 (0.72*)
3	0.91 (N=260)	0.88 (0.84*)	0.89 (0.85*)	0.66 (0.77*)
4	0.70 (N=136)	0.97 (0.80*)	0.92 (0.82*)	0.72 (0.84*)
5	0.88 (N=12)	0.90 (0.78*)	0.88 (0.80*)	0.72 (0.79*)
6	0.85 (N=84)	0.95 (0.86*)	0.88 (0.78*)	0.79 (0.81*)

Supplementary Table 1. Spatial and temporal $\delta^{18}O$-Tsurf slopes for different Antarctic sectors. Values of PD observed slope (first row, number of used data points N of MD08 data set in brackets), PD and LGM modelled slopes for the different sectors (second and third row) and of the simulated PD/LGM temporal slope (fourth row) for each sector. In the second to forth row, the number in brackets (marked with an asterisk) represent slope values calculated using precipitation-weighted mean temperatures, based on simulated daily precipitation data. The temporal slope of each sector has been calculated as the mean $\delta^{18}O$ change (LGM-PD) divided by the mean T change in the sector. Correction for glacial $\delta^{18}O$ enrichment has been applied to the LGM-PD $\delta^{18}O$ anomalies. Model results are based on the PD and LGM reference simulation.

Supplementary Note 4 – Effects of precipitation intermittency on δ^{18}O-T relations

Several studies have suggested that intermittency of Antarctic precipitation deposition might influence the isotope paleothermometer and lead to an erroneous reconstruction of past surface temperatures[33-36]. In a previous study we concluded from results of ECHAM4 simulations that such precipitation bias does not substantially bias the glacial-interglacial temperature reconstruction at Dome C[37] and Vostok[38]. Here, we reassess our former findings by re-calculating both the modern spatial as well as the LGM-PD temporal δ^{18}O-T slope, for amount-weighted mean surface temperatures. To account for the sparse and irregular occurrence of precipitation events in parts of Antarctica[12], daily values of temperature and accumulation amount are used to calculate the mean amount-weighted surface temperatures of both PD and LGM reference simulation.

For the modern spatial δ^{18}O-T slopes, we find either no substantial change or just a small decrease by precipitation intermittency for any of our 6 defined sectors (Supplementary Table 1). The average slope of all sectors is 0.82‰/°C for precipitation-weighted surface temperatures, slightly less than the slope based on arithmetic mean temperatures (m=0.90‰/°C). Similar results are found for the influence of precipitation intermittency on the simulated LGM spatial slope (arithmetic mean temperatures: 0.86‰/°C, precipitation-weighted temperatures: 0.79‰/°C). The LGM-PD temporal slopes of the different sectors are increased by 3-17% by the effect of precipitation intermittency, with the strongest local slope change in the vicinity of Vostok (21%), Dome B (21%), and Taylor Dome (26%). For Roosevelt Island, we find at unusual high slope increase of more than 40%, if intermittency is considered.

Analysing the monthly distribution of precipitation amounts (not shown), we find only a weak seasonal cycle of precipitation for most regions of Antarctica in ECHAM5-wiso. Only some coastal regions between 30°W and 150°E show relatively increased amounts of precipitation

during austral fall or winter. For the LGM climate, the absolute amount of precipitation is reduced, but the relative seasonal contributions to the total annual amount are constant in the order of ±10% as compared to the present-day contributions. This apparent stability of precipitation seasonality is one reason for the relative stability of the various δ^{18}O-T slopes in our present-day and LGM simulation.

Supplementary Note 5 – Seasonal changes of surface temperature and δ^{18}O in snow

Rather than using the modern spatial temperature-δ^{18}O relation, previous studies have also investigated the possibility of using seasonal changes in δ^{18}O and temperatures for calibrating the Antarctic isotopic thermometer (e.g., ref. 30). The basic idea behind this approach is that insolation-driven temperature changes might reflect changes of the incoming solar radiation on a seasonal or an orbital timescale in a comparable manner[31]. Supplementary Figure 4 shows for every grid point the seasonal δ^{18}O-T_{surf} slope from the simulated multi-year monthly mean values of surface temperature and δ^{18}O of precipitation. We cannot use δ^{18}O of surface snow for this analysis, as the 1-layer bucket scheme of ECHAM5-wiso does not allow simulating an annual cycle of isotope variations in the surface snow reservoir. To achieve a comparable spatial smoothing as for the calculations of the LGM-PD temporal δ^{18}O-T_{surf} slope at the different drilling locations, we then determine for every grid cell the spatial average of the neighbouring 11 x 3 (lon x lat) seasonal slopes. We find a pattern of low seasonal slopes (0.1-0.3‰/°C) in coastal regions and slightly higher slopes (0.3-0.5‰/°C) in the interior, especially in East Antarctica. Highest slopes up to 0.6‰/°C are found south of Vostok. Correlation coefficients between monthly variations of temperature and δ^{18}O in precipitation are high for most parts of Antarctica, but decrease to values of $r < 0.7$ for some coastal areas.

These seasonal slopes are substantially lower than the simulated spatial slopes both for present-day and LGM conditions (compare Fig. 5 and Supplementary Figure 4). These results are in agreement with observations by van Ommen and Morgan[30], who also report lower seasonal as compared to spatial slopes at Law Dome. Possible explanations for these lower seasonal $\delta^{18}O$-T_{surf} slopes might be warmer-than-average temperatures during snowfall events and co-variance effects[32] as well as simultaneous temperature changes at the moisture source and deposition site[30].

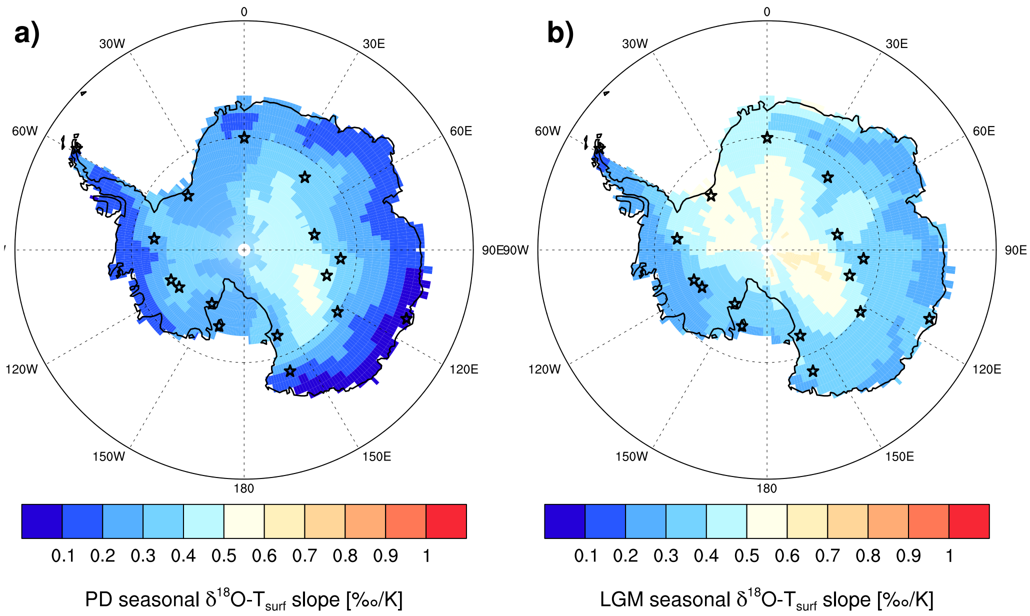

Supplementary Figure 4. Map of simulated seasonal $\delta^{18}O$-T_{surf} slopes in Antarctica. a) For the PD reference simulation. b) For the LGM reference simulation. In both plots, the seasonal $\delta^{18}O$-T_{surf} slope for every model grid point is calculated from the multi-year mean monthly values of temperature and $\delta^{18}O$ in precipitation. Symbols mark the drill position of Antarctic deep ice cores (see Table 1).

Supplementary Note 6 – Post-depositional effects: Potential isotope exchange of vapour and snow

Recent measurements of the isotopic composition of surface air above both the Greenland and Antarctic ice sheet have indicated that a non-negligible post-depositional isotope exchange between vapour and snow might occur[39-41]. Fractionation processes during sublimation, both on a diurnal and seasonal time scale, as well as isotopic exchange of firn particles and vapour trapped between the snow crystals might lead to an imprint of the isotopic composition of vapour on the $\delta^{18}O$ value of the snow. This vapour imprint could in turn alter the $\delta^{18}O$-T_{surf} relationship. ECHAM5-wiso does not explicitly account for such processes. We have therefore analysed the simulated mean distribution of $\delta^{18}O$ in near-surface water vapour ($\delta^{18}O_{vap}$) and its spatial and temporal change in relation to surface temperature. For $\delta^{18}O_{vap}$, we find much stronger depleted values over the Antarctic continent as for $\delta^{18}O$ in snow. In addition, the simulated present-day spatial $\delta^{18}O_{vap}$-T_{surf}-relation is not only shifted towards more depleted $\delta^{18}O_{vap}$ values but the slope (m = 1.40) is furthermore much stronger than the one simulated for Antarctic precipitation. Any explicit modelling of post-depositional isotope exchange between vapour and snow would likely result in a steeper simulated spatial $\delta^{18}O$-T slope for the isotopic composition of snow and thus worsen the model-data agreement. Although the potential introduction of such an additional bias cannot be taken as a direct evidence against any post-depositional vapour-snow isotope exchange, our results suggest that such exchange might not substantially alter the mean annual isotopic composition of Antarctic snow.

Supplementary Note 7 – Sensitivity tests of ECHAM5-wiso results to LGM boundary conditions

LGM ice sheet height. For further analyses of the influence of elevation on the Antarctic climate, we have studied the relationship between elevation and surface temperature, accumulation and $\delta^{18}O$ in surface snow. For the present-day, we find in the observational data set by Masson-Delmotte et al.[4] strong correlations for elevation and T_{surf} (m = -1.2°C/100m, r = 0.93, Supplementary Figure 5a) as well as for $\delta^{18}O$ (m = -1.0‰/100m, r = 0.90, Supplementary Figure 5g). These tight links are well reproduced with slightly lower values by the PD reference simulation, both for T_{surf} (m = -1.0°C/100m, r = 0.92, Supplementary Figure 5b) and $\delta^{18}O$ (m = -0.8‰/100m, r = 0.93, Supplementary Figure 5h). For the accumulation rate, the relation with elevation height appears weaker, both in the modern observations (m = -1.5cm/yr/100m, r = 0.53, Supplementary Figure 5d) and in the model results (m = -1.5cm/yr/100m, r = 0.70, Supplementary Figure 5e). These findings hold true if we look at the spatial relation between elevation, T_{surf}, accumulation, and $\delta^{18}O$ for the LGM reference simulation. Again spatial changes of temperature and isotopes are well correlated with elevation changes, but accumulation values are weaker correlated (Supplementary Figure 5b,e,h, dark blue symbols).

For the correlation of temporal elevation changes with these climate variables, we calculate the slope for each Antarctic grid cell with a prescribed LGM elevation increase of +300m or more. Such LGM-PD increases of ice sheet height lead to cooling, but the mean modelled temporal slope (m = -0.7±0.4°C/100m) is 30% lower than the simulated modern spatial slope (Supplementary Figure 5c). Large variability of the calculated slopes among the different grid cells exist, though. For the simulated LGM accumulation changes, a mean slope of -0.6±0.8cm/yr/100m is detected (Supplementary Figure 5f). For $\delta^{18}O$, the results are

comparable to temperature (strong correlation of LGM-PD $\delta^{18}O$ changes with elevation change, lower temporal slope of -0.7±0.3‰/100m, Supplementary Figure 5i), and the agreement of slopes between different grid cells is higher than for temperature. Our results indicate that corrections for past elevation effects to isotope records derived from ice cores should be done with care as temporal slopes might differ by up to 50% from the observed present-day one.

The complex interplay between LGM ice sheet height, glacial changes of GHG, ocean state and incoming solar insolation, for influencing $\delta^{18}O$ in Antarctic snowfall is further illustrated by the results of our two sensitivity experiments, which allow separating the impact of the different forcing factors (Supplementary Figure 6), if applied separately to the PD reference simulation. As expected, the massive increase of the western Antarctic ice sheet in our LGM reference simulation using the PMIP3 ice sheet reconstruction causes a major depletion in $\delta^{18}O$ at the West Antarctic ice core sites (Byrd, Siple Dome, WDC, Berkner Island, Fletcher Island). This depletion clearly outweighs at these sites the depletion caused by other LGM forcing factors. For East Antarctica, only minor LGM-PD elevation changes have been applied (see Fig. 2). Here, the influence of ice sheet height versus other glacial changes are rather equal (Dome F, Dome B), or even lower (Vostok, EDC). We also find that the applied LGM changes of ocean state, orbital parameters and GHG do not cause a uniform decrease in $\delta^{18}O$ at the different Antarctic ice core sites. Largest decreases of about 3-4‰ are simulated for Vostok, EDC, Law Dome, and Berkner Island, while smallest decreases (<1.2‰) are found for Siple Dome, Byrd and Roosevelt Island ice core sites. These differences might be explained by the non-uniform change of SST and sea ice cover, as well as regional, non-linear changes of the atmospheric circulation, induced by the change in incoming solar insolation and GHG, which in turn leads to different cooling pattern in the various regions of Antarctica.

Such non-linear behaviour might also be responsible for the modelled different $\delta^{18}O$-height slopes at WDC, Byrd, and Siple Dome (Fig. 4).

Finally, we find that the added decrease of both sensitivity experiments together results in an LGM $\delta^{18}O$ change almost equal to the one simulated in our LGM reference simulation. Synergetic processes, which only occur during a simultaneous change of ice sheet height, ocean state, orbital parameters and GHG, play apparently only a very minor role (in the order of <0.5‰) for the simulated $\delta^{18}O$ depletion at most ice core sites. Such synergetic processes could play a more important role in fully coupled atmosphere-ocean simulations, though.

LGM ocean state. In the past decades, several attempts have been conducted for reconstructing SST and sea ice cover on a global scale for the LGM period[42-44]. The GLAMAP sea surface temperatures, prescribed in our LGM reference simulation, are mainly within 1°C - 2°C of nearby MARGO values, with the exception of warmer GLAMAP than MARGO values around New Zealand[45]. However, even the latest SST reconstruction by the MARGO group is still debated and, so far, most fully-coupled atmosphere-ocean models are simulating cooler glacial (sub)tropical oceanic conditions than reconstructed[46].

In another LGM sensitivity experiment we therefore replaced the monthly GLAMAP SST and sea ice cover changes with values derived from a set of coupled ECHAM5/MPI-OM-wiso AOGCM simulations[47,48]. These simulations produced a relative cold and uniform LGM SST cooling pattern and a fair agreement between simulated sea ice concentration and proxy data[47,49]. In comparison to our PD and LGM reference simulation, a 2°-5°C stronger cooling of Pacific ocean SST and a 1°-3°C stronger cooling of Indian ocean SST are prescribed (Supplementary Figure 7a, 7b). Slightly cooler Atlantic SST are prescribed during Southern Hemisphere summer (mean of period Dec-Feb), but winter (mean of period Jun-Aug) SST changes are comparable to the GLAMAP data. Smaller regions of warmer SST up to 4°C are found at the southern tip of South America (for Dec-Feb period, only), south of Cape Agulhas

and at the western coast of Australia. In addition, warmer SST in a circumpolar belt between 55°S to 60°S are caused by a reduced Southern Hemisphere summer sea ice coverage around Antarctica in the coupled ECHAM5/MPI-OM-wiso AOGCM simulation as compared to the GLAMAP sea ice cover values (Supplementary Figure 7a). For the Southern Hemisphere winter, sea ice cover around Antarctica in this LGM sensitivity experiment is comparable to the one of our LGM reference simulation (Supplementary Figure 7b).

For the simulated LGM changes of $\delta^{18}O$ (Supplementary Figure 7c), temperature (Supplementary Figure 7d), and the spatial $\delta^{18}O$-T-relation (not shown), we find very similar results as compared to the AMIP and GLAMAP forcing. Again, the prescribed LGM PMIP3 ice sheet dominates the cooling over West Antarctica, and this sensitivity experiment also results in a much stronger cooling and $\delta^{18}O$ depletion over West Antarctica than over East Antarctica. A comparison of the simulated $\delta^{18}O$ values with the selected ice core data reveals a stronger bias of this sensitivity study, as compared to the LGM reference simulation with prescribed GLAMAP SST and sea ice data. For 9 out of the 11 selected ice cores, the model-data mismatch becomes worse, with an average absolute deviation between modelled $\delta^{18}O$ and ice core data of 1.8‰. This deviation is about 60% larger than the model bias for our LGM simulation using GLAMAP data (1.1‰). For the temporal $\delta^{18}O$-T relation, our analyses disclose a substantial difference as compared to the LGM reference simulation, too. For the majority of East Antarctic grid points, the simulated temporal $\delta^{18}O$-T slope ranges between 0.2‰/°C and 0.6‰/°C for prescribed cooler LGM SST and sea ice, as compared to the main range between 0.5‰/°C and 0.80‰/°C for prescribed GLAMAP data. No similar shift towards much lower slopes can be observed for the simulated $\delta^{18}O$-T slopes in West Antarctica. The cooler (sub)tropical SST in this sensitivity study apparently lead to a lower latitudinal temperature gradient over the ocean, which in turn leads to different water vapour sources of Antarctic precipitation. These results are in agreement with two previous studies[7,50]

also reporting lower temporal δ^{18}O-temperature slopes for weak equator-pole gradients of LGM SST changes. However, as this simulation with cooler LGM SST and sea ice boundary conditions leads to worse δ^{18}O results as the LGM simulation with GLAMAP data when compared to Antarctic ice core data, we rate the simulated lower East-Antarctic temporal δ^{18}O-T slopes of this experiment as more unlikely.

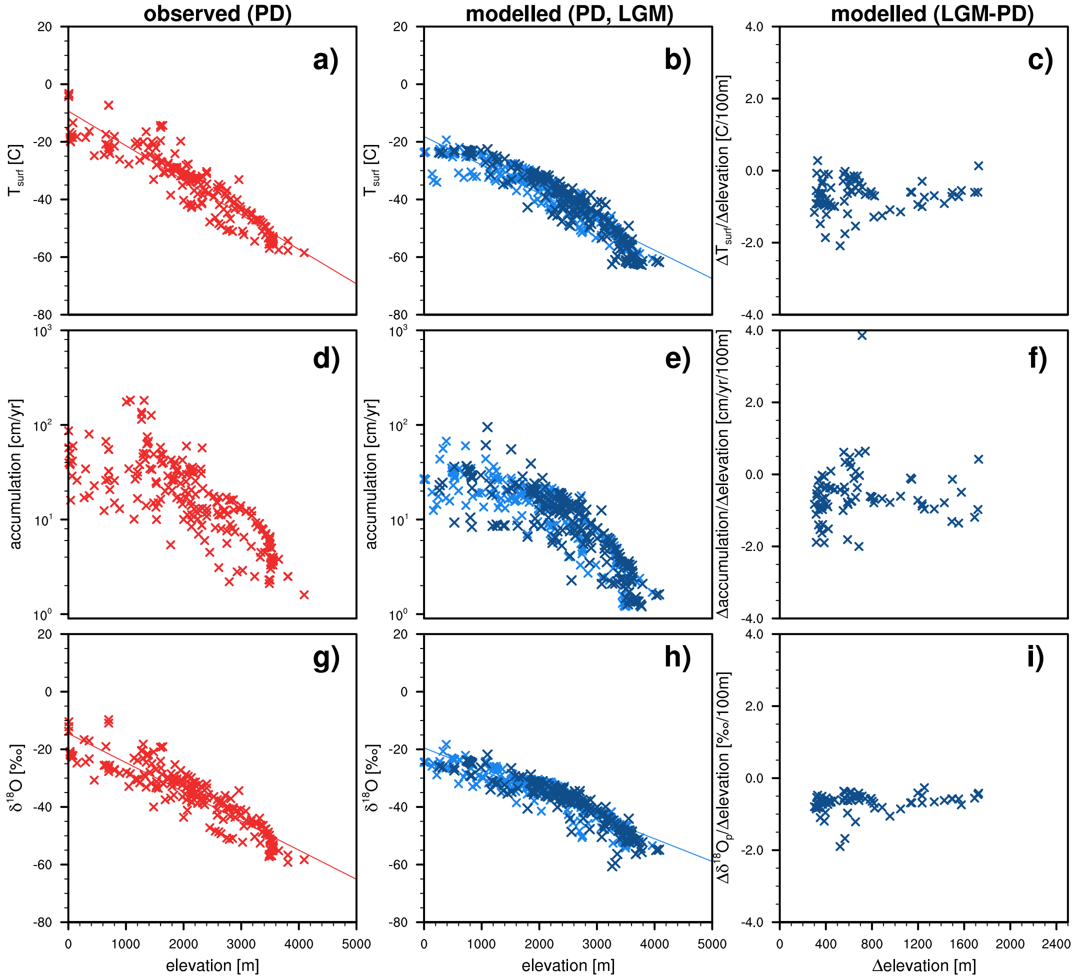

Supplementary Figure 5. Relation between prescribed ice sheet elevation and simulated temperature, accumulation, and δ^{18}O in snow. Left column: observed present-day spatial relation; middle column: simulated PD and LGM spatial relation; right column: simulated temporal slope for LGM-PD elevation changes. Simulation results are based on the PD and LGM reference simulation.

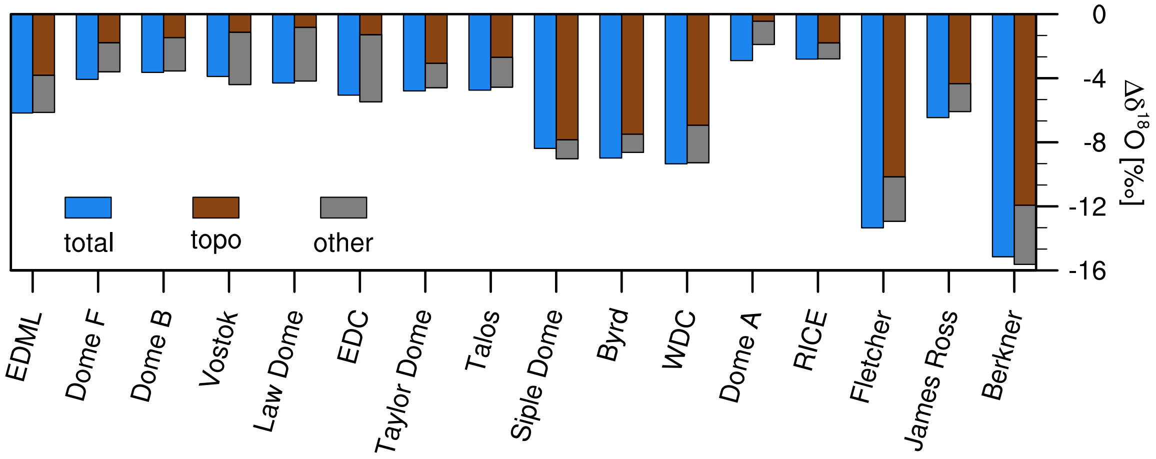

Supplementary Figure 6. Partitioning of the simulated glacial decrease in $\delta^{18}O$ in snow. The simulated total glacial decrease in $\delta^{18}O$ in snow (blue) can be separated into a partial decrease caused by glacial elevation changes (brown) versus other LGM forcing factors (grey; orbital parameters, decreased greenhouse gas concentrations, glacial SST and sea ice cover). Simulation results are based on the PD and LGM reference simulation.

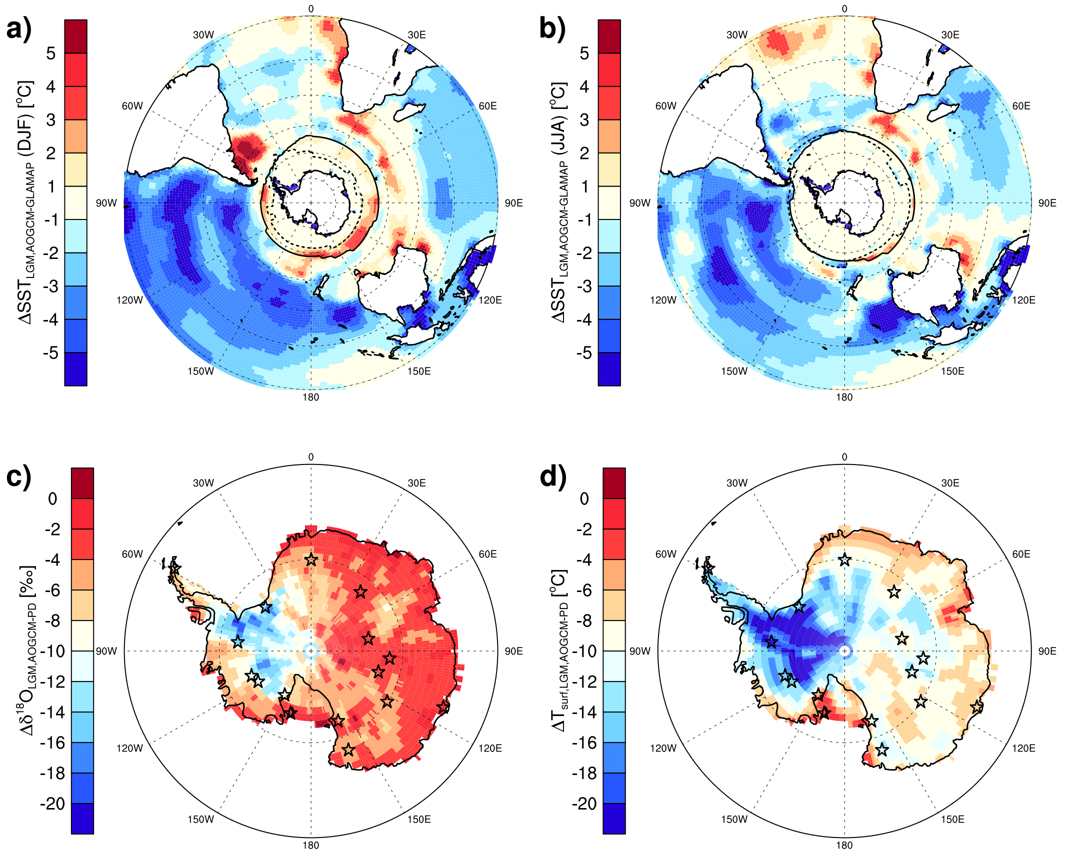

Supplementary Figure 7. Influence of SST and sea ice cover on $\delta^{18}O$ in surface snow. a) Difference of prescribed mean southern hemisphere summer (DFJ) sea surface temperatures (SST) between the sensitivity experiment, using LGM SST changes from a fully-coupled ECHAM5/MPI-OM AOGCM study, minus the LGM reference simulation, based on the GLAMAP SST data set. The solid and dashed lines indicate the 30% sea ice cover margin of the GLAMAP data set and AOGCM simulation, respectively. b) As in a) but values for the mean southern hemisphere winter (JJA). c) Map of simulated LGM-PD change of $\delta^{18}O$ in surface snow for the sensitivity experiment with prescribed SST from an ECHAM5/MPI-OM AOGCM simulation. d) As c) but for the simulated LGM-PD change of surface temperatures T_{surf}. Symbols in panel c) and d) mark the position of data from deep Antarctic ice cores, which are used for model evaluation.

Supplementary References:

1. Morgan, V. I. Antarctic Ice-Sheet Surface Oxygen Isotope Values. *J. Glaciol.* **28,** 315–323 (1982).
2. Dahe, Q., Petit, J. R., Jouzel, J. & Stiévenard, M. Distribution of Stable Isotopes in Surface Snow Along the Route of the 1990 International Trans-Antarctica Expedition. *J. Glaciol.* **40,** 107–118 (1994).
3. Giovinetto, M. B. & Zwally, H. J. Areal distribution of the oxygen-isotope ratio in Antarctica: an assessment based on multivariate models. *Annals of Glaciology* **25,** 153–158 (1997).
4. Masson-Delmotte, V. *et al.* A review of Antarctic surface snow isotopic composition: Observations, atmospheric circulation, and isotopic modeling. **21,** 3359–3387 (2008).
5. Favier, V. *et al.* An updated and quality controlled surface mass balance dataset for Antarctica. *The Cryosphere* **7,** 583–597 (2013).
6. Lee, J.-E., Fung, I., DePaolo, D. J. & Henning, C. C. Analysis of the global distribution of water isotopes using the NCAR atmospheric general circulation model. *J. Geophys. Res. Atmos.* **112,** D16306 (2007).
7. Risi, C., Bony, S., Vimeux, F. & Jouzel, J. Water-stable isotopes in the LMDZ4 general circulation model: Model evaluation for present-day and past climates and applications to climatic interpretations of tropical isotopic records. *J. Geophys. Res. Atmos.* **115,** D12118 (2010).
8. Masson-Delmotte, V. *et al.* Past and future polar amplification of climate change: climate model intercomparisons and ice-core constraints. *Clim Dyn* **26,** 513–529 (2006).
9. IPCC. *Climate Change 2013: The physical science basis. Contribution of Working Group I to the Fith Assessment Report of the Intergovernmental Panel on Climate Change* (eds Stocker, T. F. et al.). (Cambridge University Press, 2013).
10. Krinner, G., Genthon, C., Li, Z. X. & LeVan, P. Studies of the Antarctic climate with a stretched-grid general circulation model. *J. Geophys. Res. Atmos.* **102,** 13731–13745 (1997).
11. Ekaykin, A. Meteorological regime of central Antarctica and its role in the formation of isotope composition of snow thickness. (Faculté de géographie de Saint Pétersbourg, 2003).
12. Fujita, K. & Abe, O. Stable isotopes in daily precipitation at Dome Fuji, East Antarctica. *Geophys. Res. Lett.* **33,** L18503 (2006).
13. Frezzotti, M. *et al.* Spatial and temporal variability of snow accumulation in East Antarctica from traverse data. *J. Glaciol.* **51,** 113–124 (2005).
14. Grieger, J., Leckebusch, G. C. & Ulbrich, U. Net Precipitation of Antarctica: Thermodynamical and Dynamical Parts of the Climate Change Signal. *J. Clim.* **29,** 907–924 (2016).
15. Uotila, P., Lynch, A. H., Cassano, J. J. & Cullather, R. I. Changes in Antarctic net precipitation in the 21st century based on Intergovernmental Panel on Climate Change (IPCC) model scenarios. *J. Geophys. Res. Atmos.* **112,** D10107 (2007).
16. Dee, S., Noone, D., Buenning, N., Emile-Geay, J. & Zhou, Y. SPEEDY-IER: A fast atmospheric GCM with water isotope physics. *J. Geophys. Res. Atmos.* **120,** 73–91 (2015).
17. Braconnot, P. *et al.* Evaluation of climate models using palaeoclimatic data. *Nat Clim Change* **2,** 417–424 (2012).
18. Abe-Ouchi, A. *et al.* Ice-sheet configuration in the CMIP5/PMIP3 Last Glacial Maximum experiments. *Geosci. Model Dev.* **8,** 3621–3637 (2015).

19. Salamatin, A. N. *et al.* Ice core age dating and paleothermometer calibration based on isotope and temperature profiles from deep boreholes at Vostok Station (East Antarctica). *J. Geophys. Res.* **103,** 8963–8977 (1998).
20. Cuffey, K. M. *et al.* Deglacial temperature history of West Antarctica. *P Natl Acad Sci USA* **113,** 14249–14254 (2016).
21. Capron, E. *et al.* Glacial-interglacial dynamics of Antarctic firn columns: comparison between simulations and ice core air-delta N-15 measurements. *Clim. Past* **9,** 983–999 (2013).
22. Jasechko, S. *et al.* Late-glacial to late-Holocene shifts in global precipitation $\delta^{18}O$. *Clim. Past* **11,** 1375–1393 (2015).
23. Noone, D. & Sturm, C. in *Isoscapes* (eds. West, J. B., Bowen, G. J., Dawson, T. E. & Tu, K. P.) 195–219 (Springer Netherlands, 2010).
24. Werner, M., Langebroek, P. M., Carlsen, T., Herold, M. & Lohmann, G. Stable water isotopes in the ECHAM5 general circulation model: Toward high-resolution isotope modeling on a global scale. *J. Geophys. Res. Atmos.* **116,** D15109 (2011).
25. LeGrande, A. N. & Schmidt, G. A. Ensemble, water isotope-enabled, coupled general circulation modeling insights into the 8.2 ka event. *Paleoceanography* **23,** PA3207 (2008).
26. Yoshimura, K., Oki, T., Ohte, N. & Kanae, S. A quantitative analysis of short-term18O variability with a Rayleigh-type isotope circulation model. *J. Geophys. Res.* **108,** 4647 (2003).
27. Cauquoin, A. *et al.* Comparing past accumulation rate reconstructions in East Antarctic ice cores using ^{10}Be, water isotopes and CMIP5-PMIP3 models. *Clim. Past* **11,** 355–367 (2015).
28. Parrenin, F. *et al.* The EDC3 chronology for the EPICA Dome C ice core. *Clim. Past* **3,** 485–497 (2007).
29. Bazin, L. *et al.* An optimized multi-proxy, multi-site Antarctic ice and gas orbital chronology (AICC2012): 120-800 ka. *Clim. Past* **9,** 1715–1731 (2013).
30. van Ommen, T. D. & Morgan, V. I. Calibrating the Ice Core Paleothermometer Using Seasonality. *J. Geophys. Res.* **102,** 9351–9357 (1997).
31. Laepple, T. & Lohmann, G. Seasonal cycle as template for climate variability on astronomical timescales. *Paleoceanography* **24,** PA4201 (2009).
32. Sime, L. C., Lang, N., Thomas, E. R., Benton, A. K. & Mulvaney, R. On high-resolution sampling of short ice cores: Dating and temperature information recovery from Antarctic Peninsula virtual cores. *J. Geophys. Res.* **116,** D20117 (2011).
33. Noone, D. & Simmonds, I. Implications for the interpretation of ice-core isotope data from analysis of modelled Antarctic precipitation. *Annals of Glaciology* **27,** 398–402 (1998).
34. Jouzel, J. *et al.* Magnitude of isotope/temperature scaling for interpretation of central Antarctic ice cores. *J. Geophys. Res. Atmos.* **108,** 4361 (2003).
35. Masson-Delmotte, V. *et al.* A comparison of the present and last interglacial periods in six Antarctic ice cores. *Clim. Past* **7,** 397–423 (2011).
36. Sime, L. C. & Wolff, E. W. Antarctic accumulation seasonality. *Nature* **479,** E1–E2 (2011).
37. Jouzel, J. *et al.* Orbital and millennial Antarctic climate variability over the past 800,000 years. *Science,* **317,** 793–796 (2007).
38. Werner, M., Heimann, M. & Hoffmann, G. Isotopic composition and origin of polar precipitation in present and glacial climate simulations. *Tellus Ser. B-Chem. Phys. Meteorol.* **53,** 53–71 (2001).

39. Steen-Larsen, H. C. *et al*. Continuous monitoring of summer surface water vapor isotopic composition above the Greenland Ice Sheet. *Atmos. Chem. Phys.* **13,** 4815–4828 (2013).
40. Casado, M. *et al*. Continuous measurements of isotopic composition of water vapour on the East Antarctic Plateau. *Atmos. Chem. Phys.* **16,** 8521–8538 (2016).
41. Ritter, F. *et al*. Isotopic exchange on the diurnal scale between near-surface snow and lower atmospheric water vapor at Kohnen station, East Antarctica. *The Cryosphere* **10,** 1647–1663 (2016).
42. CLIMAP Project Members. Seasonal reconstruction of the Earth surface at the last glacial maximum. *Map Chart Series* **MC-36** (Geological Society of America, Boulder, Colorado, 1981).
43. Schäfer-Neth, C. & Paul, A. Gridded global LGM SST and salinity reconstruction. *IGBP PAGES/World Data Center for Paleoclimatology Data Contribution Series* **#2003-046** (Boulder CO, USA, 2003).
44. MARGO Project Members. Constraints on the magnitude and patterns of ocean cooling at the Last Glacial Maximum. *Nature Geoscience* **2,** 127–132 (2009).
45. Sime, L. C. *et al*. Southern Hemisphere westerly wind changes during the Last Glacial Maximum: model-data comparison. *Quaternary Sci Rev* **64,** 104–120 (2013).
46. Hargreaves, J. C., Paul, A., Ohgaito, R., Abe-Ouchi, A. & Annan, J. D. Are paleoclimate model ensembles consistent with the MARGO data synthesis? *Clim. Past* **7,** 917–933 (2011).
47. Zhang, X., Lohmann, G., Knorr, G. & Xu, X. Different ocean states and transient characteristics in Last Glacial Maximum simulations and implications for deglaciation. *Clim. Past* **9,** 2319–2333 (2013).
48. Werner, M. *et al*. Glacial–interglacial changes in $H_2^{18}O$, HDO and deuterium excess – results from the fully coupled ECHAM5/MPI-OM Earth system model. *Geosci. Model Dev.* **9,** 647–670 (2016).
49. Abelmann, A. *et al*. The seasonal sea-ice zone in the glacial Southern Ocean as a carbon sink. *Nature Communications* **6,** 8136 (2015).
50. Lee, J.-E., Fung, I., DePaolo, D. J. & Otto-Bliesner, B. Water isotopes during the Last Glacial Maximum: New general circulation model calculations. *J. Geophys. Res.* **113,** D19109– (2008).
