# Supplementary material for: Reconciling glacial Antarctic water stable isotopes with ice sheet topography and the isotopic paleothermometer
Source: Nat Commun. 2018 Aug 30;9:3537. doi: 10.1038/s41467-018-05430-y (PMC6117368; doi:10.1038/s41467-018-05430-y)
Supplement: Supplementary file 2 — Reviewers' comments [file 41467_2018_5430_MOESM2_ESM.pdf]

Reviewers' comments:

Reviewer #1 (Remarks to the Author):

Summary:

This is a very good paper. And its importance and originality is clearly sufficiently high to merit publication in Nat Comms. It is of broad appeal to ice core, paleoclimate, climate modelling, and ice sheet scientists. The investigation/paper is sound, it is well thought out, the statistics are robust, and the description is generally good. I do however think the current emphasis in the abstract (and the title) should be changed. This would enable it be more influential across many fields.

Please see below for my more detailed comments.

Major points:

The abstract and title concentrate on the match of the spatial gradient and the temporal gradient for LGM->Holocene change. This is probably a chance result, given it is known that spatial gradient and the temporal gradients do not match for many other time periods, places, and climatic shifts. An emphasis on other more interesting and robust results would also make the paper's importance to a wider range of paleoclimate scientist much clearer. In particular, the results on LGM SSTs, WAIS surface elevation changes, and the impact of model resolution changes are much more interesting (compared to those on the paleothermometer). The abstract and title should reflect this.

The LGM simulation is performed using updated PMIP3 glacial boundary condition files. It would be useful to know to what extent the updated boundary condition files, versus the improved model physics/resolution impact on the results. A set of simulations which explores the difference between the older Antarctic (PMIP2) ice sheet configuration, and the new updated one, would be particularly helpful for this.

Minor comments:
P1L17 "pronounced glacial West Antarctic.." rephrase, perhaps a "surprisingly large West…"
P2L68 a "warm bias of approximately 5oC" suggests that that the authors might slightly tone down a little about the excellence of the simulated climate in places in the text…
P4L75 following on from above, "ability to provide extremely satisfying". Whilst I do not, of course, doubt the ability of Werner et al to provide an extremely satisfying simulation, perhaps this could be rephrased?
L85 New paragraph?
P5L102"have documented" rephrase, perhaps something like "indicate" instead
L103 "in the order of" replace, perhaps "of"
L109-110 "in good to very good agreement". What does this mean? Better to quantify, within p/m *.* permil. This is repeated on L212-213
L113 quote model-data differences as average bias, and RMSE values?
L119 caused by improved model "physics" rather than "skill"?
L128-132 Could a separate table of model versus ice core derived accumulation changes be of use?
L133 Clarify that its "The LGM to preindustrial/Holocene isotopic paleothermometer."
L133-> Mostly the words spatial or temporal "slope" are used, but occasionally "gradient" slips in. I would tend to use "gradient". Anyway, consistency.
L164 "These results support the general idea that isotope-temperature relations should be used for a reconstruction of precipitation-weighted mean temperature.." Is this not only true if spatial

gradients were also calculated using precipitation-weighted temperature – which is not generally the case. Perhaps remove or rephrase?
L173"multiplying"?
L180 clarify that, whilst the spatial gradient and the temporal gradient mostly match for LGM->Holocene change, there are other published simulation results that suggest that over other timescales, climatic events, and time periods these spatial and temporal gradients do not match. Include references.
L187 onwards. The results on LGM SSTs, WAIS surface elevation changes, and the impact of model resolution changes are (arguably) more interesting than those on the paleothermometer. Perhaps the abstract should be rewritten to reflect these?
L223 "on a smaller sector by sector scale" it perhaps should be noted that the spatial gradient is fundamentally dependent on the choice of the spatial region. For example, see Sime et al 2008.
L234-5 "Our simulations results might help to anticipate and better understand d18O changes for some of the upcoming…" this is written rather weakly. Rephrase or remove. At the moment this doesn't say anything useful..
Table 1: would be nice to include the elevation of the sites. Given this is a rather complicated table, perhaps splitting into two could be beneficial for clarity?
Supplementary info:
L29 "have erroneously high accumulation values." What does this mean?
L188-189 odd phrasing: "maps the situation"
L298 odd phrasing: "in a considerable manner"
L319 "Eat Antarctica".

Reviewer #2 (Remarks to the Author):

This manuscript reports an impressive advance in the modeling of precipitation isotopes in Antarctica. There has not previously been a model analysis that performed so well against measured constraints of temperature and isotopes, and in the context of such a wealth of information from ice cores.
I think this study would be a good contribution for Nature Comm., as it connects a number of important processes and observations of interest to the glaciology, paleoclimate, climate, and meteorology communities.

I do not support publication in the present form, however. The presentation seems to be misleading in places and founded in incomplete analysis of one key issue.

1. The Abstract claims that the model is "capable of correctly simulating the modern Antarctic climate...," while the manuscript reveals there is a 5 degree warm bias. The bias is not uniform but grows from zero to its full amount in the range of -20 to -35 C surface temperatures. Whether this bias should be regarded as unimportant depends on its cause and what performance is required of the model. Calling it "correct" does not seem right.

2. Similarly, the manuscript makes clear, in contrast to general statements in the Abstract and elsewhere, that the LGM-to-present isotope changes are systematically overcalculated in West Antarctica and undercalculated in East Antarctica. The incorrect difference between model results for these two regions is about 3 per mille, which seems like a large number in this context!
It appears to be more than half of the difference in "topo" components of the isotopic changes (Figure S7).

3. The claim is made that "our model results support the LGM ice sheet reconstruction of Antarctica proposed by the PMIP3 consortium" (line 216+), which specifies a surface elevation in central West Antarctica of more than 900m higher than present. Such a much thicker ice sheet (> 1 km thicker, given isostatic effects) is inconsistent with geologic data (cosmogenic exposure ages on nunataks) and all ice sheet models driven by climate histories (Pollard and DeConto, Golledge, etc.), so this is a potentially very interesting contribution to the debate.
I am certain, however, that this hypothesis is not consistent with the temperature history at the WDC site, which I published last year (Cuffey, K.M., G.D. Clow, and 7 others (2016). Proc. Nat. Acad. Sci. 113(50), 14249–14254), and which is Ref. 26 of the Supplement in the submitted paper. Here is the problem: An ice sheet thinning from LGM to Holocene (a time of increasing accumulation rate) must be accomplished by increased flux divergence, which impels a greater vertical velocity downward and hence cooling of the ice at depth. The greater the thinning, the greater this cooling effect. This, in turn, means that the measured borehole temperature signal implies a smaller LGM to present climatic surface temperature change (i.e., the fraction of the observed cold zone in the borehole that can be ascribed to the deglacial climate warming decreases as the assumed thinning increases). The calculation of this effect is shown explicitly in the Supplement of my PNAS paper (see Figure S3 therein). Specifying a 1.2 km thinning (required for a 900 m elevation decrease) raises the reconstructed surface temperature change by around 3 C, relative to the best-guess value of 11.3 C. Such an 8 C surface temperature change is well below the 12 to 20 C range calculated in the submitted paper.
The authors seem not to have understood this effect, as they want to dismiss the WDC analysis by saying the thickness change is too small, whereas in fact the larger the thickness change, the less their own results agree with the WDC reconstruction. They can contact me directly by email if they want clarification. Given this problem, plus the systematic over-prediction of West vs. East Antarctic isotope changes in the submitted paper, I wonder if the authors' analysis is actually proving that the PMIP3 thickness changes are too large. And, perhaps, simultaneously disproving a scenario of zero thickness change, so the answer is something in the mid-range.

--Kurt Cuffey

Reviewer #3 (Remarks to the Author):

Review of “Reconciling the validity of the isotopic paleothermometer in Antarctica” by Werner et al.

As an overall comment, I find the manuscript presented by Werner and co-workers is of real scientific interest even though the sensitivity experiments and the discussion of the experiment design could be improved. I have real doubts however that it fits the scope of Nature Communications as stated on the website: “Papers published by the journal represent important advances of significance to specialists within each field”. In the comments below, I highlight that the manuscript is, in my reading, a very incremental study and does not present a real breakthrough in our understanding of the field.

The manuscript presents a series of numerical experiments with the latest generation of the atmospheric only ECHAM model enhanced with water isotopes for Antarctica. The experiments presented cover present-day and LGM climates and a few sensitivity experiments to the LGM boundary conditions.

The text is clear, well presented. The quality of the discussion and of the experiments performed allows to follow the arguments presented very easily at a superficial level. For a specialist, understanding thoroughly the assumptions made in forcing the atmosphere only model requires to make frequent jumps forward and backward to the Supplementary Information which is also relatively easy to follow – that is when one has found the correct paragraph in the long additional text provided (34 pages on my computer !!!). For me this is already a hint that perhaps the manuscript in its present format is not presented to best advantage in the Nature Communications format.

The message conveyed by the manuscript is not very new or revolutionary. It is mainly built as a repeat of previous water isotope enabled simulations for Antarctica and discussion about its performance regarding ice-core records – and its relevance to paleo-estimations of temperature change in Antarctica. The subject in itself is of importance since it bears lots of implications to the well-known polar amplification of climate change, a subject of much discussions around the IPCC assessments. By proposing a 15% error bar on the computation of temperature from ice-core isotopic records, the authors are updating our knowledge to current climate models standards. It is a useful information for the community.
Clearly, this is a very incremental study. It is acknowledged by the authors themselves in the manuscript, see for example lines 180-186. Even though I do not underestimate the necessary work put into updating the water isotope cycle in a new generation atmospheric model, it is not by itself bringing any fundamentally new message when put in perspective with what was already published on the topic. It is certainly refining the error bars, but the overall same message could have been written with the previous generation of the ECHAM model. The cause for improvement of the resulting d18Op fields for the LGM over the previous version is not well explained (cf. Lines 254-258), such that other groups running atmospheric water isotopic enabled models have no chance to use the present study to understand how to improve their model representation over Antarctica. What is the main driving process of the improvements? The reader would have benefited from a discussion to comparable studies with other models and their relative complexities in the representation of key processes.
As such, I hence find difficult to find the “important advances” requested by the manuscript type. It certainly shows that having a higher spatial resolution does help in resolving the precipitation patterns and change in Antarctica. However, this is not in itself a game changer either since a) it has been continuously argued over the past years and b) the model still presents some important biases in particular in temperature estimation at the surface over the ice-sheet.

Regarding the setup of the simulations, the choice of AMIP style experiments should be discussed. The group of the first author has published already coupled simulations with the same atmospheric model and hence have the potential for analyzing in details the effect of all their model experiments design. The proposed simulation with SST only from the coupled model comes short of that target. Indeed, there is no comparable experiment presented for the present-day state nor any analysis of the choices made for the d18Osw, of major interest here. The decision of using present-day distribution from the GISS, known to have large errors, and add a 1 per mil shift for glacial conditions is surprising to say the least. Especially when the authors could derive alternatives using relationships derived from their coupled simulations. Along the same line, the use of the GLAMAP surface conditions should be discussed in particular in the light of a) the MARGO reconstruction and b) the apparent sensitivity to the SSTs found in their sensitivity experiments.

The manuscript presented is at its strongest, I think, when looking at sensitivity experiments around the LGM state. The additional experiments with an – incomplete factor separation analysis – are certainly of interest to disentangle the different processes at play. The authors could have made it even more interesting by computing some maximum / minimum bounds for the topographical change in Western Antarctica so as for the simulated d18Oice to have stayed within the observational range. This would have lead to a much stronger conclusion with important

consequences for the groups trying to reconstruct the changes in Antarctic ice-sheet altitude. The statement that it works with PMIP3 is very short: what is the error margin on the altitude change? Considering that the ice-sheet used in PMIP3 is a merged ice-sheet constructed from several reconstructions, this is quite crucial.

As a conclusion, I feel that the manuscript would be better targeted in a more specialized journal with larger space within the text to include all the information required to fully understand the work presented. I have no doubt however that the content itself is of high quality and should be published to the benefit of the paleoclimate science community.

Response to Reviewers

We thank all three reviewers for their thoroughly and very helpful comments. We have addressed them in the revised manuscript in the following manner (reviewers' comments are printed in blue, our answers and taken actions in black):

Reviewer #1 (Remarks to the Author):

Summary:

This is a very good paper. And its importance and originality is clearly sufficiently high to merit publication in Nat Comms. It is of broad appeal to ice core, paleoclimate, climate modelling, and ice sheet scientists. The investigation/paper is sound, it is well thought out, the statistics are robust, and the description is generally good. I do however think the current emphasis in the abstract (and the title) should be changed. This would enable it be more influential across many fields.

We thank Reviewer #1 for this positive and encouraging assessment.

Please see below for my more detailed comments.

Major points:

The abstract and title concentrate on the match of the spatial gradient and the temporal gradient for LGM->Holocene change. This is probably a chance result, given it is known that spatial gradient and the temporal gradients do not match for many other time periods, places, and climatic shifts. An emphasis on other more interesting and robust results would also make the paper's importance to a wider range of paleoclimate scientist much clearer. In particular, the results on LGM SSTs, WAIS surface elevation changes, and the impact of model resolution changes are much more interesting (compared to those on the paleothermometer). The abstract and title should reflect this.

We have changed the title and rewritten the abstract. It now puts more emphasis on the role of the various boundary conditions as well as on the results of the additionally performed simulations with different ice sheet reconstructions (see below).

The LGM simulation is performed using updated PMIP3 glacial boundary condition files. It would be useful to know to what extent the updated boundary condition files, versus the improved model physics/resolution impact on the results. A set of simulations which explores the difference between the older Antarctic (PMIP2) ice sheet configuration, and the new updated one, would be particularly helpful for this.

Based on these recommendations we have expanded our study by several new LGM simulations. Among others, we have replaced the prescribed PMIP3 glacial boundary conditions by (i) the older PMIP2 data set (i.e. the ICE-5G reconstruction), (ii) the very recently published PMIP4 data sets (i.e. ICE-6G_C and GLAC-1D reconstruction). The effect of prescribed different glacial boundary conditions on the simulated isotope signal in Antarctica is now discussed in detail in the revised manuscript.

We have also performed two new simulations (PD and LGM in coarse T31 spectral resolution) to quantify the effect of different model resolution on the simulated isotope signal in Antarctica. The text part which compares our simulation results to an older ECHAM4 study has been updated, accordingly.

Please note:
All line numbers of our answers refer to the revised manuscript and supplement version.

Minor comments:

P1L17 “pronounced glacial West Antarctic..” rephrase, perhaps a “surprisingly large West…”

Changed by rephrasing the whole abstract.

P2L68 a “warm bias of approximately 5oC” suggests that that the authors might slightly tone down a little about the excellence of the simulated climate in places in the text…

We have rephrased relevant text parts to follow this recommendation.

P4L75 following on from above, “ability to provide extremely satisfying”. Whilst I do not, of course, doubt the ability of Werner et al to provide an extremely satisfying simulation, perhaps this could be rephrased?

Done. (L79-81)

L85 New paragraph?

Done. (L88)

P5L102”have documented” rephrase, perhaps something like “indicate” instead

Done. (L105)

L103 “in the order of” replace, perhaps “of”

Done. (L106)

L109-110 “in good to very good agreement”. What does this mean? Better to quantify, within p/m *.* permil. This is repeated on L212-213

The text has been rephrased (L118) and expanded by the discussion of different ice sheet configurations (L135-142).

L113 quote model-data differences as average bias, and RMSE values?

Model-data differences and RMSE values are now given in the new Table 2. RMSE values are discussed on L135-142.

L119 caused by improved model “physics” rather than “skill”?

Done. (The comparison to older ECHAM4 results is now described in more detail on L171ff.)

L128-132 Could a separate table of model versus ice core derived accumulation changes be of use?

We prefer to list the accumulation rates in the text, only, as they are not used in the following text part which evaluates the isotopic paleothermometer. But we have added the elevation of the drilling sites in Table 1 and split it into two tables (new Table 1 and Table 3) for clarity reasons (as recommend further below). Furthermore, the ice core entries in the tables have been reordered. The sites are now listed clockwise in longitudinal direction.

L133 Clarify that its “The LGM to preindustrial/Holocene isotopic paleothermometer.”

Done. (L205)

L133-> Mostly the words spatial or temporal “slope” are used, but occasionally “gradient” slips in. I would tend to use “gradient”. Anyway, consistency.

We prefer to use the word “slope” and now consistently use it in the whole manuscript.

L164 “These results support the general idea that isotope-temperature relations should be used for a reconstruction of precipitation-weighted mean temperature..” Is this not only true if spatial gradients were also calculated using precipitation-weighted temperature – which is not generally the case. Perhaps remove or rephrase?

Yes, the statement is also true for spatial gradients and we have rephrased the text, accordingly. (L236-239)

L173”multiplying”?

The text has been rephrased. (L246-248)

L180 clarify that, whilst the spatial gradient and the temporal gradient mostly match for LGM->Holocene change, there are other published simulation results that suggest that over other timescales, climatic events, and time periods these spatial and temporal gradients do not match. Include references.

Done. (L262-265)

L187 onwards. The results on LGM SSTs, WAIS surface elevation changes, and the impact of model resolution changes are (arguably) more interesting than those on the paleothermometer. Perhaps the abstract should be rewritten to reflect these?

Done (see our answer to the first major comment above).

L223 “on a smaller sector by sector scale” it perhaps should be noted that the spatial gradient is fundamentally dependent on the choice of the spatial region. For example, see Sime et al 2008.

Done. (L279-280)

L234-5 “Our simulations results might help to anticipate and better understand d18O changes for some of the upcoming…” this is written rather weakly. Rephrase or remove. At the moment this doesn’t say anything useful..

We have rephrased this statement. (L291-292)

Table 1: would be nice to include the elevation of the sites. Given this is a rather complicated table, perhaps splitting into two could be beneficial for clarity?

We have included the elevation of the drilling sites in Table 1 and split it into two tables (new Table 1 and Table 3) for clarity reasons.

Supplementary info:
L29 “have erroneously high accumulation values.” What does this mean?

We have explained this in more detail in the revised supplement. (L26-27)

L188-189 odd phrasing: “maps the situation”

We have rephrased this statement. (L172-173)

L298 odd phrasing: “in a considerable manner”

Rephrased. (L274-275)

L319 “Eat Antarctica”.

Corrected. (The whole paragraph has been reformulated and moved to the main manuscript.)

Reviewer #2 (Remarks to the Author):

This manuscript reports an impressive advance in the modeling of precipitation isotopes in Antarctica. There has not previously been a model analysis that performed so well against measured constraints of temperature and isotopes, and in the context of such a wealth of information from ice cores.

I think this study would be a good contribution for Nature Comm., as it connects a number of important processes and observations of interest to the glaciology, paleoclimate, climate, and meteorology communities.

We thank Reviewer #2 for this overall positive and encouraging assessment.

I do not support publication in the present form, however. The presentation seems to be misleading in places and founded in incomplete analysis of one key issue.

1. The Abstract claims that the model is "capable of correctly simulating the modern Antarctic climate...," while the manuscript reveals there is a 5 degree warm bias. The bias is not uniform but grows from zero to its full amount in the range of -20 to -35 C surface temperatures. Whether this bias should be regarded as unimportant depends on its cause and what performance is required of the model. Calling it "correct" does not seem right.

The abstract has been rewritten, following the various suggestions of all three reviewers.

2. Similarly, the manuscript makes clear, in contrast to general statements in the Abstract and elsewhere, that the LGM-to-present isotope changes are systematically overcalculated in West Antarctica and undercalculated in East Antarctica. The incorrect difference between model results for these two regions is about 3 per mille, which seems like a large number in this context! It appears to be more than half of the difference in "topo" components of the isotopic changes (Figure S7).

This systematic bias of modelled LGM-to-present isotope changes in East and West Antarctica is now discussed in more detail in the revised manuscript (see also our answer to the next comment).

3. The claim is made that "our model results support the LGM ice sheet reconstruction of Antarctica proposed by the PMIP3 consortium" (line 216+), which specifies a surface elevation in central West Antarctica of more than 900m higher than present. Such a much thicker ice sheet (> 1 km thicker, given isostatic effects) is inconsistent with geologic data (cosmogenic exposure ages on nunataks) and all ice sheet models driven by climate histories (Pollard and DeConto, Golledge, etc.), so this is a potentially very interesting contribution to the debate.
I am certain, however, that this hypothesis is not consistent with the temperature history at the WDC site, which I published last year (Cuffey, K.M., G.D. Clow, and 7 others (2016). Proc. Nat. Acad. Sci. 113(50), 14249–14254), and which is Ref. 26 of the Supplement in the submitted paper. Here is the problem:
An ice sheet thinning from LGM to Holocene (a time of increasing accumulation rate) must be accomplished by increased flux divergence, which impels a greater vertical velocity downward and hence cooling of the ice at depth. The greater the thinning, the greater this cooling effect. This, in turn, means that the measured borehole temperature signal implies a smaller LGM to present climatic surface temperature change (i.e., the fraction of the observed cold zone in the borehole that can be ascribed to the deglacial climate warming decreases as the assumed thinning increases). The calculation of this effect is shown explicitly in the Supplement of my PNAS paper (see Figure S3 therein). Specifying a 1.2 km thinning (required for a 900 m elevation decrease) raises the reconstructed surface

temperature change by around 3 C, relative to the best-guess value of 11.3 C. Such an 8 C surface temperature change is well below the 12 to 20 C range calculated in the submitted paper.
The authors seem not to have understood this effect, as they want to dismiss the WDC analysis by saying the thickness change is too small, whereas in fact the larger the thickness change, the less their own results agree with the WDC reconstruction. They can contact me directly by email if they want clarification.
Given this problem, plus the systematic over-prediction of West vs. East Antarctic isotope changes in the submitted paper, I wonder if the authors' analysis is actually proving that the PMIP3 thickness changes are too large. And, perhaps, simultaneously disproving a scenario of zero thickness change, so the answer is something in the mid-range.

We thank the reviewer for this very helpful comment. In response to these remarks as well as the other two reviews we have expanded our study by several alternate LGM simulations. Among others, we have replaced the prescribed PMIP3 glacial boundary conditions by (i) the older PMIP2 data set (i.e. the ICE-5G reconstruction), (ii) the very recently published PMIP4 data sets (i.e. ICE-6G_C and GLAC-1D reconstruction). The effect of prescribed different glacial boundary conditions on the simulated isotope signal in Antarctica is now analyzed and discussed in detail in the revised manuscript.

For West Antarctica, we find indeed that a smaller LGM ice sheet height does lead to an improved simulation of the isotope changes at WDC, Byrd and Siple Dome drilling site. Based on our new model results and measured ice core delta O-18 changes, we estimate the potential height changes these sites. The findings are reported in the revised manuscript, and the abstract has been changed accordingly.

Reviewer #3 (Remarks to the Author):

Review of “Reconciling the validity of the isotopic paleothermometer in Antarctica” by Werner et al.

As an overall comment, I find the manuscript presented by Werner and co-workers is of real scientific interest even though the sensitivity experiments and the discussion of the experiment design could be improved. I have real doubts however that it fits the scope of Nature Communications as stated on the website: “Papers published by the journal represent important advances of significance to specialists within each field”. In the comments below, I highlight that the manuscript is, in my reading, a very incremental study and does not present a real breakthrough in our understanding of the field.

The manuscript presents a series of numerical experiments with the latest generation of the atmospheric only ECHAM model enhanced with water isotopes for Antarctica. The experiments presented cover present-day and LGM climates and a few sensitivity experiments to the LGM boundary conditions.

The text is clear, well presented. The quality of the discussion and of the experiments performed allows to follow the arguments presented very easily at a superficial level. For a specialist, understanding thoroughly the assumptions made in forcing the atmosphere only

model requires to make frequent jumps forward and backward to the Supplementary Information which is also relatively easy to follow – that is when one has found the correct paragraph in the long additional text provided (34 pages on my computer !!!). For me this is already a hint that perhaps the manuscript in its present format is not presented to best advantage in the Nature Communications format.

We thank Reviewer #2 for this careful assessment of our manuscript. Following the comments of all reviewers, we have rephrased and changed the order of several text parts for clarity reasons. This enabled us to shorten the supplemental text and omit one supplemental figure.

The message conveyed by the manuscript is not very new or revolutionary. It is mainly built as a repeat of previous water isotope enabled simulations for Antarctica and discussion about its performance regarding ice-core records – and its relevance to paleo-estimations of temperature change in Antarctica. The subject in itself is of importance since it bears lots of implications to the well-known polar amplification of climate change, a subject of much discussions around the IPCC assessments. By proposing a 15% error bar on the computation of temperature from ice-core isotopic records, the authors are updating our knowledge to current climate models standards. It is a useful information for the community.
Clearly, this is a very incremental study. It is acknowledged by the authors themselves in the manuscript, see for example lines 180-186.

Our former results (discussed on lines 180-186 in the previously submitted manuscript version) analyzed the validity of the modern analogue hypothesis for different drilling sites of the East Antarctic ice sheet (Vostok, EDC), only. To our knowledge, our new analyses are the most comprehensive approach so far to disentangle the interpretation of ice core 18O records in temperature and ice sheet elevation changes, with consideration of regional variations from all available records. Given the different characteristics of the isotopic records in West and East Antarctica during the last glacial and deglaciation (e.g. WAIS Divide Project Members, Nature, 2013) we rate our findings as novel and highly interesting for the community. We have rephrased the relevant text passage.

Furthermore, following the comments of all reviewers, our revised manuscript has been expanded by several additional LGM simulations to study the influence of different Antarctic ice sheet reconstructions on the isotopic signal in precipitation. Our results indicate that the PMIP3 reconstruction results in the highest agreement with the various ice core records, but most likely overestimates ice sheet changes in West Antarctica. However, the most recent ice sheet reconstructions suggested for the upcoming PMIP4 experiments (Kageyama et al., Clim.Past, 2017) don't yield improved results but seem to underestimate past elevation changes in this region. Again, we are not aware of any comparable study on this topic and rate our results as highly novel.

Even though I do not underestimate the necessary work put into updating the water isotope cycle in a new generation atmospheric model, it is not by itself bringing any fundamentally new message when put in perspective with what was already published on the topic. It is certainly refining the error bars, but the overall same message could have been written with the previous generation of the ECHAM model. The cause for improvement of the resulting d18Op fields for the LGM over the previous version is not well explained (cf. Lines 254-258),

such that other groups running atmospheric water isotopic enabled models have no chance to use the present study to understand how to improve their model representation over Antarctica. What is the main driving process of the improvements?

For the revised manuscript we have also performed new PD and LGM simulations with a coarser (T31) model resolution. These new simulations allow us to disentangle the various reasons of the reported improvements of ECHAM5-wsio. The results are explained in the revised manuscript (L171-192 of the revised manuscript).

The reader would have benefited from a discussion to comparable studies with other models and their relative complexities in the representation of key processes.

We agree that such discussion would be very valuable. However, to our knowledge no comparable studies with other isotope models exist. One comparison between the performance of different isotope models form LGM-late Holocene changes was published by Jasechko et al. (Clim.Past, 2015). For Antarctica, a large inter-model spread of simulated glacial δ^{18}O changes was reported in this study, but not further discussed regarding any representation of key processes in the different models. We discuss the study of Jasechko et al. in the revised supplement, L147-163.

As such, I hence find difficult to find the “important advances” requested by the manuscript type. It certainly shows that having a higher spatial resolution does help in resolving the precipitation patterns and change in Antarctica. However, this is not in itself a game changer either since a) it has been continuously argued over the past years and b) the model still presents some important biases in particular in temperature estimation at the surface over the ice-sheet.

We agree that the importance of model resolution has been discussed in previous studies, already (among others, by ourselves in Werner et al., JGR, 2011). We also agree that our new ECHAM5-wiso simulations are not perfect and discuss the existing temperature bias in the revised manuscript (revised manuscript, L71-73, L213-215, supplement L40-46).

In line with the reviewer’s comment, we neither rate only an increased model resolution nor an improved model performance alone as an “important advance”, which would qualify our manuscript for publication in Nature Communications. However, we think that both achievements (as compared to previous studies) are indispensable to have high confidence in the validity of our presented results.

As already mentioned above, we rate both the consistent analyses of isotopic changes in East and West Antarctica as well as the (now included) comparison of different LGM ice sheet reconstructions as highly novel and a clear advancement to previous studies.

Regarding the setup of the simulations, the choice of AMIP style experiments should be discussed. The group of the first author has published already coupled simulations with the same atmospheric model and hence have the potential for analyzing in details the effect of all their model experiments design. The proposed simulation with SST only from the coupled model comes short of that target. Indeed, there is no comparable experiment presented for the present-day state nor any analysis of the choices made for the d18Osw, of major interest

here. The decision of using present-day distribution from the GISS, known to have large errors, and add a 1 per mil shift for glacial conditions is surprising to say the least. Especially when the authors could derive alternatives using relationships derived from their coupled simulations.

In Werner et al. (GMD, 2016) we compare in detail the simulated d18Osw of our fully-coupled isotope GCM setup with the present-day distribution from the GISS data set. We reported regional differences in the order of ±0.25‰ on a global scale. Due to the current limitation of the GISS data set (in terms of both spatial and temporal coverage) we don't think it is possible to clearly attribute these differences to either measurement or model deficits.

For the LGM, our coupled isotope GCM simulations indeed show non-uniform changes of the glacial d18Osw enrichment. We simulate a globally averaged mean increase of +0.84‰ as compared to the modern climate, but more positive LGM d18Osw anomalies of up to +1.5‰ exist in the ACC region (see discussion of Fig. 10a in Werner et al., GMD, 2016).

As a first-order estimate, a change of prescribed d18Osw values will directly lead to a change of d18O in Antarctic precipitation of approximately equal size (Jouzel et al., JGR, Vol 108, D12, 2003). Thus, we expect just an overall minor change of our simulated d18O values in Antarctica, if we replace the GISS data set (with a uniform +1‰ shift for the LGM) with PD and LGM d18Osw values from our coupled simulations. Larger d18O changes for individual ice core sites might be possible, though.

To explicitly test this influence of the d18Osw boundary condition, we have performed an additional simulation for PD and LGM, each, where we have replaced the GISS d18Osw values by the modeled d18Osw distribution, reported in Werner et al., 2016. The difference in the prescribed d18Osw values is shown in the following maps (left: PD, right: LGM).

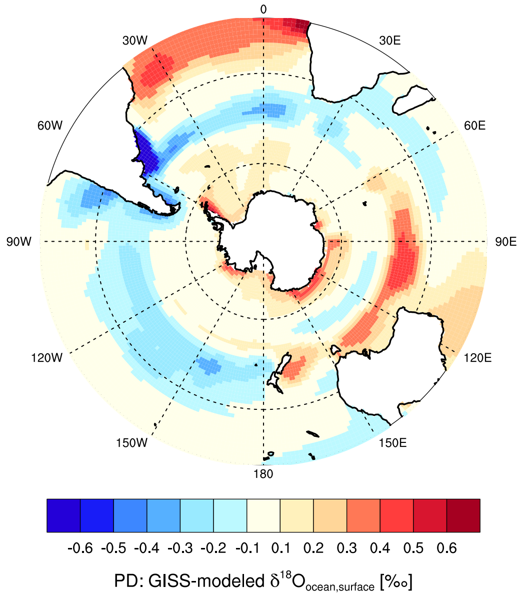

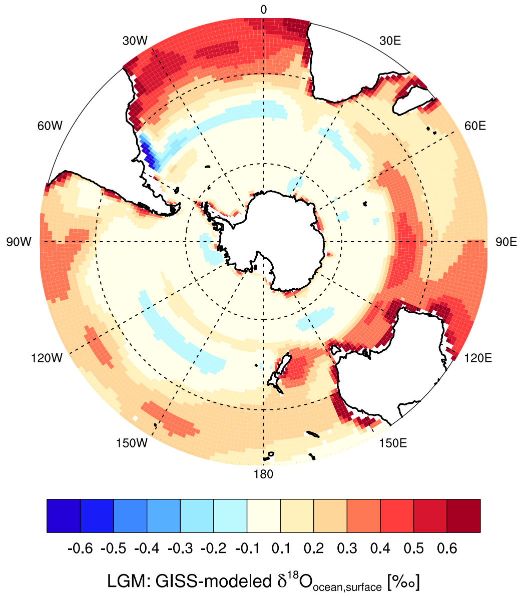

These different d18Osw boundary conditions lead to simulated changes of d18O in Antarctic precipitation of 0‰ to +0.2‰ for both the PD and LGM climate. Accordingly, the difference

in the simulated LGM-PD d18O changes in precipitation of our reference simulation (using GISS data) and the simulations with prescribed modeled d18Osw values is also minor. It is shown in the following map:

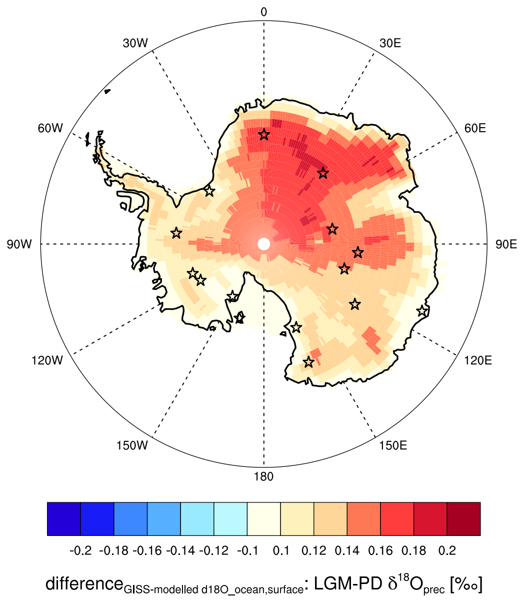

Using GISS data instead of our modeled d18Osw values leads to a mean extra enrichment of the LGM-PD d18O_prec anomalies at the selected ice core sites of +0.12‰. Largest changes are detected for Dome F (+0.18‰) and smallest changes for Law Dome (+0.04‰). These small changes do not alter our analyses results regarding the different temporal and spatial d18O-T-slopes. Thus, we rate the choice of the d18sw boundary condition as noncritical. The results of these additional sensitivity tests are mentioned in the revised manuscript (L156-160).

For the setup of the sensitivity run with SST changes from our coupled simulation, we have added simulated LGM SST anomalies (derived from the control and LGM coupled simulation, reported in Werner et al., GMD, 2016) to the SST of our PD reference simulation. For this reason, no extra experiment for the present-day state is presented in the manuscript.

Along the same line, the use of the GLAMAP surface conditions should be discussed in particular in the light of a) the MARGO reconstruction and b) the apparent sensitivity to the SSTs found in their sensitivity experiments.

The choice of GLAMAP SST and sea ice cover, a comparison to the latest MARGO reconstruction as well as to our sensitivity experiment with ocean boundary conditions from a coupled ECHAM5/MPIOM simulation has been discussed in Supplement Section S7 of our manuscript. We have expanded this discussion in the revised text version, and key results of this comparison are now mentioned in the main text (L161-167).

The manuscript presented is at its strongest, I think, when looking at sensitivity experiments around the LGM state. The additional experiments with an – incomplete factor separation analysis – are certainly of interest to disentangle the different processes at play. The authors

could have made it even more interesting by computing some maximum / minimum bounds for the topographical change in Western Antarctica so as for the simulated d18Oice to have stayed within the observational range. This would have lead to a much stronger conclusion with important consequences for the groups trying to reconstruct the changes in Antarctic ice-sheet altitude. The statement that it works with PMIP3 is very short: what is the error margin on the altitude change? Considering that the ice-sheet used in PMIP3 is a merged ice-sheet constructed from several reconstructions, this is quite crucial.

We thank the reviewer for this very helpful comment. In response to these remarks as well as the other two reviews we have expanded our study by several alternate LGM simulations. Among others, we have replaced the prescribed PMIP3 glacial boundary conditions by (i) the older PMIP2 data set (i.e. the ICE-5G reconstruction), (ii) the very recently published PMIP4 data sets (i.e. ICE-6G_C and GLAC-1D reconstruction). The effect of prescribed different glacial boundary conditions on the simulated isotope signal in Antarctica is now analyzed and discussed in detail in the revised manuscript.

As a conclusion, I feel that the manuscript would be better targeted in a more specialized journal with larger space within the text to include all the information required to fully understand the work presented. I have no doubt however that the content itself is of high quality and should be published to the benefit of the paleoclimate science community.

We hope that the applied changes and additional analyses are in line with all suggested changes and comments and that this revised manuscript version is now rated as suitable for publication in Nature Communication by Reviewer #3, too.

REVIEWERS' COMMENTS:

Reviewer #1 (Remarks to the Author):

I find this version of the manuscript much improved, particularly the inclusion of additional results from the new simulations: it is now a much stronger paper. I would though still like to see two minor changes to the text:

L320 'it is noteworthy that our results are in contrast to previous studies that suggest a larger mismatch of the spatial and temporal slopes for warmer climates in Antarctica'...

This is an odd phrasing ref30 showed that a LGM->PD paleothermometer value approx matches that usually quoted for the spatial relationship. So, really this study shows the same as ref30 results (see Fig 4, cold climate crosses). I suggest rephasing this to make it clear that whilst various studies, using various isotope-enabled models, have found that an approx match between the commonly quoted spatial relationship and the LGM-> PD temporal relationship, there is evidence that this likely may not hold for other climate shifts e.g. LIG->PD.

Further, it might also be worth noting that the spatial relationship does seem to depend (very strongly) on the selection of the spatial domain (ref29, Figure 5). The authors could also check again L361->L371 for any similar issues.

Reviewer #2 (Remarks to the Author):

I think this manuscript is now ready to publish. It fits well with the mission of Nature Comms., as it presents an important new contribution while including quite a few issues deserving argumentation. I affirm the comments in my original review, and also judge that the authors have done an adequate job responding to referees. I want to point out that there is an alternative interpretation to the findings in this paper. The authors' essentially argue that large Antarctic elevation changes are required to make model isotope and temperature results conform with observational constraints. As an alternative, perhaps the problem is that the climate model under-calculates the effects of climate forcings and feedbacks on LGM Antarctic climate. This would be a great topic of discussion.

Reviewer #3 (Remarks to the Author):

This is my second review of "Reconciling glacial-interglacial changes of Antarctic water stable isotopes, ice sheet topography, and the isotopic paleothermometer" by Dr. Werner et al. To cut straight to my overall impression, I think that the authors have done an impressive job in revising the manuscript. The amount of analysis and hence the quality of the resulting manuscript has been much improved. Well done and thanks. I recommend publication.

I still have my doubts as to whether a manuscript that has more than half of its length in the supplementary really fits the proposed format, but I leave to the editor of Nature Comms. to decide whether this is appropriate for the journal.

I have a few mostly minor comments:

1/ Page 4 line 83: "The model skill is key for our confidence ..." => I think it should be "The model skill for present-day is key TO our confidence ..."

2/ Page 4, line 85: "Main focus ..." => "The main focus ..."

3/ Page line 87: ""as well as different pattern" => plural/singular inconsistency "as well as [a] different pattern[s]" (choose one from the brackets)

4/ Page 93: I do not really understand why the record of James Ross is not considered. The argument that it is small and not well represented is weak. At ~1°x1° many features are not well represented in details. Also if it close to the ocean, it should have an oceanic-type signal and hence represent a larger area that should be comparable to the model geographical scale. I would include it whatever is shows, out of completeness. The reader would not have then the impression that something is hidden.

5/ Page 10, line 245 and in other instances I think, please refer to the actual paragraph in the supplement. Your supplement is fully numbered, so this is an easy task to do.

Response to Reviewers

Again, we thank all three reviewers for their thoroughly reading and positive evaluation of our manuscript. We have addressed their comments in this revised manuscript in the following manner (reviewers' comments are printed in blue, our answers and taken actions in black):

Reviewer #1 (Remarks to the Author):

I find this version of the manuscript much improved, particularly the inclusion of additional results from the new simulations: it is now a much stronger paper. I would though still like to see two minor changes to the text:

L320 'it is noteworthy that our results are in contrast to previous studies that suggest a larger mismatch of the spatial and temporal slopes for warmer climates in Antarctica'...

This is an odd phrasing ref30 showed that a LGM->PD paleothermometer value approx matches that usually quoted for the spatial relationship. So, really this study shows the same as ref30 results (see Fig 4, cold climate crosses). I suggest rephasing this to make it clear that whilst various studies, using various isotope-enabled models, have found that an approx match between the commonly quoted spatial relationship and the LGM-> PD temporal relationship, there is evidence that this likely may not hold for other climate shifts e.g. LIG->PD.

The statement has been rephrased.

Further, it might also be worth noting that the spatial relationship does seem to depend (very strongly) on the selection of the spatial domain (ref29, Figure 5). The authors could also check again L361->L371 for any similar issues.

This dependency is already mentioned in the revised manuscript (L284): "It should be noted though that the sector scale agreement might depend on the choice of the spatial region[29]".

Reviewer #2 (Remarks to the Author):

I think this manuscript is now ready to publish. It fits well with the mission of Nature Comms., as it presents an important new contribution while including quite a few issues deserving argumentation. I affirm the comments in my original review, and also judge that the authors have done an adequate job responding to referees. I want to point out that there is an alternative interpretation to the findings in this paper. The authors' essentially argue that large Antarctic elevation changes are required to make model isotope and temperature results conform with observational constraints. As an alternative, perhaps the problem is that the climate model under-calculates the effects of climate forcings and feedbacks on LGM Antarctic climate. This would be a great topic of discussion.

We agree with the reviewer than such alternative interpretation could also explain our results. However, given the overall good performance of our model for the present-day

climate and its general agreement with both observational data and other LGM model results (see Supplementary Note 2), we rate this alternative as less likely.

Reviewer #3 (Remarks to the Author):

This is my second review of "Reconciling glacial-interglacial changes of Antarctic water stable isotopes, ice sheet topography, and the isotopic paleothermometer" by Dr. Werner et al. To cut straight to my overall impression, I think that the authors have done an impressive job in revising the manuscript. The amount of analysis and hence the quality of the resulting manuscript has been much improved. Well done and thanks. I recommend publication.

I still have my doubts as to whether a manuscript that has more than half of its length in the supplementary really fits the proposed format, but I leave to the editor of Nature Comms. to decide whether this is appropriate for the journal.

I have a few mostly minor comments:

1/ Page 4 line 83: "The model skill is key for our confidence ..." => I think it should be "The model skill for present-day is key TO our confidence ..."

Corrected.

2/ Page 4, line 85: "Main focus ..." => "The main focus ..."

Corrected.

3/ Page line 87: ""as well as different pattern" => plural/singular inconsistency "as well as [a] different pattern[s]" (choose one from the brackets)

Corrected.

4/ Page 93: I do not really understand why the record of James Ross is not considered. The argument that it is small and not well represented is weak. At ~1°x1° many features are not well represented in details. Also if it close to the ocean, it should have an oceanic-type signal and hence represent a larger area that should be comparable to the model geographical scale. I would include it whatever is shows, out of completeness. The reader would not have then the impression that something is hidden.

Model results for the James Ross drilling site are now included in the manuscript.

5/ Page 10, line 245 and in other instances I think, please refer to the actual paragraph in the supplement. Your supplement is fully numbered, so this is an easy task to do.

The Supplementary Notes have been rearranged and we now refer to all of them in the manuscript.
